# Supplementary material for: The Transcriptional and Epigenomic Foundations of Ground State Pluripotency
Source: Cell. 2012 Apr 27;149(3):590–604. doi: 10.1016/j.cell.2012.03.026 (PMC3398752; doi:10.1016/j.cell.2012.03.026)
Supplement: Document S1. Article plus Supplemental Information [file mmc7.pdf]

Cell

The Transcriptional and Epigenomic Foundations of Ground State Pluripotency

Hendrik Marks,[1] Tüzer Kalkan,[2] Roberta Menafra,[1] Sergey Denissov,[1] Kenneth Jones,[2] Helmut Hofemeister,[5] Jennifer Nichols,[2,3] Andrea Kranz,[5] A. Francis Stewart,[5] Austin Smith,[2,4,*] and Hendrik G. Stunnenberg[1,*]
[1]Department of Molecular Biology, Faculty of Science, Nijmegen Centre for Molecular Life Sciences (NCMLS), Radboud University, PO Box 9101, 6500 HB Nijmegen, The Netherlands
[2]Wellcome Trust Centre for Stem Cell Research and Stem Cell Institute
[3]Department of Physiology, Development and Neuroscience
[4]Department of Biochemistry
University of Cambridge, Tennis Court Road, Cambridge CB2 1QR, UK
[5]Genomics, BioInnovationsZentrum, Technische Universität Dresden, Am Tatzberg 47-51, D-01307 Dresden, Germany
*Correspondence: austin.smith@cscr.cam.ac.uk (A.S.), h.stunnenberg@ncmls.ru.nl (H.G.S.)
DOI 10.1016/j.cell.2012.03.026

SUMMARY

Mouse embryonic stem (ES) cells grown in serum exhibit greater heterogeneity in morphology and expression of pluripotency factors than ES cells cultured in defined medium with inhibitors of two kinases (Mek and GSK3), a condition known as "2i" postulated to establish a naive ground state. We show that the transcriptome and epigenome profiles of serum- and 2i-grown ES cells are distinct. 2i-treated cells exhibit lower expression of lineage-affiliated genes, reduced prevalence at promoters of the repressive histone modification H3K27me3, and fewer bivalent domains, which are thought to mark genes poised for either up- or downregulation. Nonetheless, serum- and 2i-grown ES cells have similar differentiation potential. Precocious transcription of developmental genes in 2i is restrained by RNA polymerase II promoter-proximal pausing. These findings suggest that transcriptional potentiation and a permissive chromatin context characterize the ground state and that exit from it may not require a metastable intermediate or multilineage priming.

INTRODUCTION

Mouse embryonic stem (ES) cells are characterized by the potency to generate all somatic and germline lineages in vitro and in chimaeric embryos (Smith, 2001). The nature of the transcriptional and epigenetic machinery that maintains this potential throughout massive in vitro expansion has been the subject of intense investigation (Young, 2011). Interest is further heightened by appreciation that knowledge of the molecular underpinning of mouse ES cells may enable derivation of equivalent human naive pluripotent stem cells (Hanna et al., 2010).

ES cells are described as transcriptionally hyperactive (Efroni et al., 2008). Promiscuous transcription has been suggested to constitute a platform for lineage specification (Loh and Lim, 2011). When taken together with the observation that several pluripotency factors are expressed heterogeneously (Chambers et al., 2007; Niwa et al., 2009; Toyooka et al., 2008), the notion has arisen that pluripotency may inherently be a metastable condition (Graf and Stadtfeld, 2008; Hanna et al., 2009; Hayashi et al., 2008). Attention has also been drawn to colocalization at many promoters of histone 3 lysine 4 trimethylation (H3K4me3), associated with transcriptional activation, and histone 3 lysine 27 trimethylation (H3K27me3), linked with repression (Azuara et al., 2006; Bernstein et al., 2006; Stock et al., 2007). These bivalent domains are posited to be poised for either up- or downregulation and to provide an epigenetic blueprint for lineage determination.

The preceding views are based on analyses of ES cells cultured in serum and therefore subject to uncontrolled multifactorial perturbations. It is now possible to derive and maintain pluripotent mouse ES cells without serum factors by using two small molecule kinase inhibitors (2i) in combination with the cytokine leukemia inhibitory factor (LIF) (Ying et al., 2008). The 2i inhibitors, PD0325901 and CHIR99021, selectively target mitogen-activated protein kinase kinase (Mek) and glycogen synthase kinase-3 (Gsk3), respectively. The inhibitors shield pluripotent cells from differentiation triggers: fibroblast growth factor-4 stimulation of the Mek-Erk pathway and endogenous repressor activity of Tcf3 (Kunath et al., 2007; Wray et al., 2011). Use of 2i has enabled derivation of germline-competent ES cells from all mouse strains tested and for the first time from rats (Buehr et al., 2008; Hanna et al., 2009; Kiyonari et al., 2010; Li et al., 2008; Nichols et al., 2009). 2i thus provides a better-tuned environment for rodent ES cells. Indeed, the mosaic expression of pluripotency factors observed in serum is effectively eliminated in 2i (Wray et al., 2011). Furthermore, culture in 2i may mimic the environment in the mature mouse inner cell mass (ICM) where the fibroblast growth factor receptor is downregulated in the epiblast (Guo et al., 2010).

Here we applied massively parallel sequencing technology to characterize the global transcriptome and to map selected histone modifications in naive mouse ES cells maintained in 2i compared with heterogeneous cultures in serum.

RESULTS

Transcriptome Analysis

Three ES cell lines derived and maintained in 2i plus LIF ("2i" ES cells) were compared with three ES cell lines established and cultured in serum plus LIF ("serum" ES cells) (Tables S1 and S2). Each cell line is functionally pluripotent as demonstrated by competence to generate high-contribution chimaeras with germline transmission. Expression values from RNA-seq data were calculated by quantifying the number of sequence reads for each gene with standardized RPKM values (reads per kilobase of exon model per million mapped sequence reads). This comparison showed that 1,489 genes have more than 2-fold higher transcript abundance in 2i (p value < 0.2), whereas 1,947 genes exhibit more than 2-fold higher expression in serum (Figure 1A). Moreover, 160 genes expressed in 2i (RPKM > 0.5) were silent in serum (RPKM < 0.2) and 461 genes were expressed only in serum (Figure S1A and Table S3 available online). The majority of categorized stem cell maintenance genes ([SCM] GO:0019827), including validated core pluripotency factors, *Pou5f1*, *Nanog*, *Sox2*, *Esrrb*, *Klf2*, *Klf4*, and *Tbx3*, are transcribed to similar levels in 2i and serum (Figure 1B and Figure S1B). Nine SCM genes are more highly expressed in 2i (Figure 1B and Figure S1C). Of these, only *Tcl1* has been implicated as a regulator of self-renewal (Ivanova et al., 2006), and although this transcript is more abundant in 2i, it is also well-expressed in serum. In serum, 16 SCM genes showed higher expression. Factors in this group, such as c-Myc and the Id proteins, are known to be induced by Erk signaling and by serum. They may confer additional robustness to the pluripotent state to counter differentiation stimuli (Ying et al., 2003a). Interestingly, several of these genes are induced when ICM cells are explanted in medium containing serum (Figure S1D; Tang et al., 2010).

Functional annotation clustering of differentially expressed genes by Gene Ontology ([GO] PANTHER [protein analysis through evolutionary relationships]) and Pathway (KEGG [Kyoto encyclopedia of genes and genomes]) analysis (Figure 1C) revealed that genes upregulated in 2i are highly enriched for terms associated with metabolic processes. This is probably in part a direct consequence of inhibition of Mek and Gsk3 and the absence of serum constituents. Reduced c-Myc may further affect metabolic networks. Major differences are also apparent for genes involved in cell-cycle regulation. Proliferation is similar in the two conditions (Ying et al., 2008), however, reflecting the absence of G1 restriction in ES cells.

Upregulated genes in serum are significantly enriched for GO terms linked to developmental processes, particularly ectoderm and mesoderm germ layer specification (Figure 1C). Genes such as *Pax6*, *T (Brachyury)*, and *Runx1* show very low or undetectable expression in 2i but appreciable transcription in serum (Figure 1D). Other ectoderm and mesoderm marker genes such as *Runx3*, *Sox18*, *Cdx4*, and *Tal1* are also activated in serum, although only to low levels. In contrast, several genes associated with the germline or with endoderm are expressed at similar levels in both conditions.

ES cells maintained in 2i are morphologically uniform and rather homogeneous in expression of pluripotency regulators (Wray et al., 2010). In contrast, serum ES cells are heterogeneous in morphology (Figure S2A) and expression of factors such as Nanog, Rex1, Stella, and Klf4 (Chambers et al., 2007; Hayashi et al., 2008; Toyooka et al., 2008; Figure S2B). In serum, ES cells with a mono-allelic green fluorescent protein (GFP) knockin at the Rex1 (*Zfp42*) locus comprise GFP-positive and -negative populations that can be separated by flow cytometry (Figure S2C; Wray et al., 2011). These populations are functionally distinct. Rex1GFP-positive cells plated in serum generate colonies of undifferentiated cells, whereas Rex1GFP-negative cells produce predominantly small, differentiated colonies (Figure S2D). On plating in 2i, the yield of ES cell colonies in Rex1-positive cells is more than 10 times that in Rex1-negative cells. Rex1-negative cells have therefore largely lost clonogenic self-renewal capacity. Consistent with this, although they express Oct4, they lack Nanog and Klf4 (Figure S2E). All Nanog-positive and almost all Klf4-positive cells are within the Rex1GFP-positive fraction. Expression is still mosaic within this population, reflecting transcriptional fluctuation (Chambers et al., 2007; Kalmar et al., 2009; Figure S2E). In contrast, ES cells in 2i are almost uniformly positive for Rex1, Klf4, and Nanog (Wray et al., 2010; Figure S2B). We examined the transcriptome of the Rex1-positive fraction in serum and found a similar increased expression of a subset of SCM genes as in bulk ES cells (Figure 1B). Some ectoderm- and mesoderm-associated genes were also upregulated compared with expression in 2i ES cells but others showed little induction (Figure 1D). In general, differentiation genes were more highly expressed in Rex1GFP-low cells (Figures S2F–S2H). Nonetheless, transcriptomes of the Rex1-positive compartment in serum show many differences from 2i ES cells (Figure S2I). Notably, many genes that show higher expression in 2i are not upregulated in Rex1-positive serum ES cells (Figure 1B). Therefore it is unlikely that a subpopulation of serum cells persist in an equivalent state to 2i.

Transcriptome Interconversion

To assess whether the distinct gene expression patterns in 2i and serum represent "fixed" transcriptional states, we transferred 2i cells to serum and vice versa. Within two to three passages, cultures adopted morphological characteristics of the new condition. We carried out RNA-seq analyses after eight passages. Most of the SCM genes that showed lower expression in 2i were upregulated in serum, whereas transcripts elevated in 2i were downregulated (Figure 2A red dots and black squares, respectively). Reciprocal behavior was observed when serum cells were transferred to 2i (Figure 2B). Furthermore, genes involved in ectoderm and mesoderm germ layer specification were broadly upregulated after transfer of 2i cells to serum and the reverse transcriptional changes were observed when serum cells were passaged in 2i (Figures 2A and 2B, blue stars). Typical examples are shown in Figure 2C. Irrespective of the direction of exchange between 2i and serum, 818 genes were expressed more highly (>2-fold) in 2i and 1,209 showed higher levels in serum (Figure 2D). GO classification identified developmental genes and cell-cycle control genes as highly enriched upon transfer to serum, whereas genes upregulated in 2i were mainly associated with metabolic categories (Figure 2E). The reciprocity in transcriptome changes demonstrates that the transcriptional profiles are interconvertible.

A

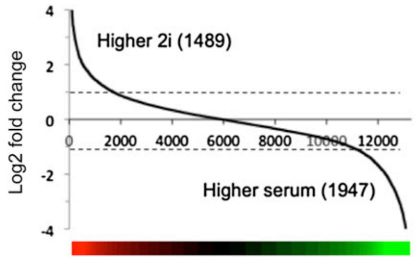

B

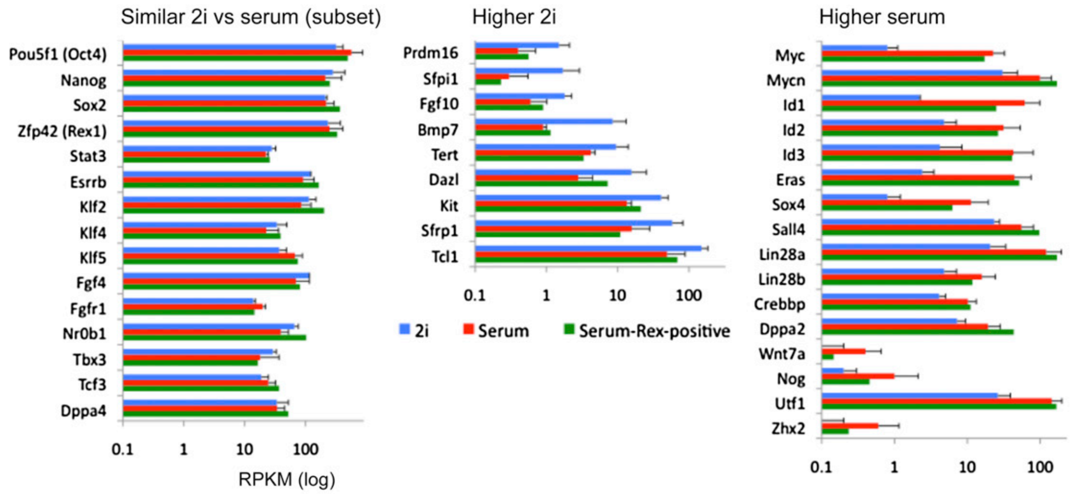

C

Higher 2i

PANTHER biological processes

Term	Count	Total category	P-Value
Lipid, fatty acid and steroid metabolism	86	780	8.7E-08
Fatty acid metabolism	28	192	4.0E-05
Carbohydrate metabolism	56	552	2.0E-04
Immunity and defense	131	1594	2.5E-04
Other carbon metabolism	14	79	9.0E-04
Other metabolism	54	562	9.6E-04
Detoxification	14	83	1.4E-03
Phospholipid metabolism	19	140	2.1E-03
Cation transport	43	464	6.3E-03

KEGG pathway

Term	Count	Total category	P-Value
Lysosome	32	119	9.5E-12
Propanoate metabolism	10	30	8.1E-05
Tryptophan metabolism	10	40	8.6E-04
Hematopoietic cell lineage	15	84	9.4E-04
Glycosphingolipid biosynthesis	6	14	1.4E-03
Glutathione metabolism	11	52	1.6E-03

Higher serum

PANTHER biological processes

Term	Count	Total category	P-Value
Developmental processes	293	2106	8.8E-14
Mesoderm development	95	533	7.1E-10
Ectoderm development	105	659	4.3E-08
Cell structure and motility	145	1015	1.1E-07
Neurogenesis	91	579	7.1E-07
Synaptic transmission	49	261	3.4E-06
Cell proliferation and differentiation	117	881	6.7E-05
Protein phosphorylation	89	657	2.9E-04
JNK cascade	16	60	3.0E-04
Cell cycle control	57	390	7.0E-04
Muscle development	24	133	2.6E-03
MAPKKK cascade	28	181	9.6E-03
Cell cycle	98	877	3.9E-02

KEGG pathway

Term	Count	Total category	P-Value
Pathways in cancer	73	323	4.8E-12
Focal adhesion	51	198	1.1E-10
ECM-receptor interaction	25	83	6.4E-07
MAPK signaling pathway	50	265	6.2E-06
TGF-beta signaling pathway	21	87	2.0E-04
Wnt signaling pathway	29	149	4.8E-04
Hedgehog signaling pathway	15	54	5.0E-04

D

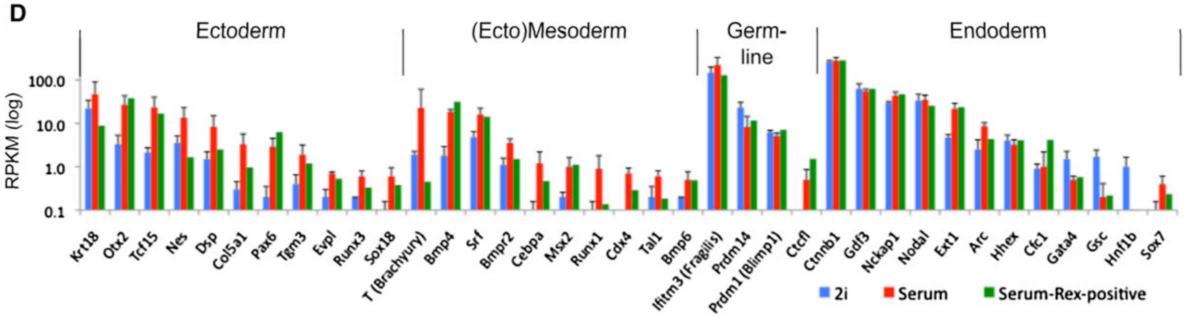

Histone Modification Profiles

We performed chromatin immunoprecipitation and deep sequencing (ChIP-seq) to analyze posttranslational histone modifications: H3K4me3 and H3K36me3 associated with active promoters and transcribed genes, respectively; H3K27me3 linked to silencing, and H3K9me3 associated with constitutive heterochromatin and imprinted genes (Table S1). We also analyzed the polycomb repressor complex 2 (PRC2) component Ezh2 that methylates H3K27 (Cao et al., 2002).

Determination of average profiles over 2,000 genes that are most highly expressed in both conditions (Figure 3A and Figure S3A) revealed conventional distribution of H3K4me3 on active promoters and of H3K36me3 extending over the coding body. The H3K9me3 ChIP-seq state maps of 2i and serum cells were nearly identical. In both conditions deposits were most prominent at satellites and imprinted genes (Figures S3B–S3E). As expected, the H3K27me3 mark is absent from actively transcribed loci (Figure 3B). It appears to be mutually exclusive with H3K36me3 (Figures 3A–3C and Figure S3F), in line with recent biochemical data showing that PRC2 activity is inhibited by active marks including H3K36me3 (Schmitges et al., 2011). However, H3K27me3 is widely deposited over intergenic regions and inactive genes at levels appreciably higher than random distribution. This lawn of H3K27me3 is qualitatively and quantitatively similar in 2i and serum ES cells (Figure 3C). A pronounced difference is apparent only at promoters of lowly expressed genes (Figure 3B). The averaged profile of these promoters showed markedly less H3K27me3 in 2i than in serum. This was not reflected in any overall increase in expression (Figure S3H). Three independent 2i ES cell lines exhibited a significantly reduced level of H3K27me3 at the promoters of poorly or nonexpressed genes compared to the level in serum cultures (Figure 3D). To investigate whether differences in H3K27me3 deposition reflected heterogeneity in serum, we performed H3K27me3 ChIP-seq on Rex1-positive and Rex1-negative serum subpopulations. Intriguingly, the H3K27me3 signals were very similar, each resembling the total serum ES cell population (Figure 3E).

Intensity plots covering a region of 5 kb up- and downstream of all promoters that are decorated with H3K27me3 in serum demonstrate the major reduction in H3K27me3 in 2i cells (Figure 3F). Ezh2 levels were also diminished at these locations in 2i. In either condition, the H3K27me3 pattern follows a camelback profile with a depleted region around the transcriptional start site. Ezh2 appears as a single peak centered on this trough. Representative examples of differential H3K27me3 profiles are shown in Figure 3G (Figure S3I shows the PCR validation). The *Gata6*, *Pax9*, and *Lhx1* genes are barely expressed in either 2i or serum, but in all cases the H3K27me3 signal around the promoter is selectively and greatly reduced in 2i. For the *Lhx1* locus, adjacent *Aatf* provides a contrasting example of a gene that is productively transcribed in both 2i and serum and remains devoid of H3K27me3 in either condition.

Given the interchangeable transcriptome profiles between 2i and serum, we examined the epigenomic landscape in ES cells transferred between the two conditions. Cells taken from 2i into serum acquired substantially elevated H3K27me3 at H3K27me3-associated promoters (Figure 3H), the *Hox* clusters (Figure S4A), and many other loci (Figure S4B). Conversely, serum ES cells transferred into 2i displayed diminished H3K27me3 at these loci. Ezh2 and Suz12 localization similarly switched between culture conditions (Figure 3H and Figures S4A and S4B). Therefore these epigenomic states are interconvertible.

H3K27me3 was reduced by between 63%–75% over all *Hox* clusters in 2i (Figure 3G and Figure S4A). The *Hoxc* locus follows this pattern but with a distinctive variation; in the *Hoxc13-c12* region H3K27me3 deposition is lost entirely. This region (boxed in Figure 3G) is transcribed only in 2i. Strand-specific RNA-seq profiling after rRNA depletion revealed two nonoverlapping transcripts on the reverse and forward strand (both boxed in Figure S4C, left). These ncRNAs are distinct from the HOTAIR ncRNA located between *HOXC11-12* in human (Rinn et al., 2007). Consistent with recent findings (Guttman et al., 2010), we detected known as well as multiple previously unidentified ncRNAs. Many of these, such as H19, showed differential expression between 2i and serum (Figure S4C and Table S4).

Global Redistribution of H3K27me3

We computed the number of H3K27me3 reads over nonrepetitive regions and plotted the frequency of occurrence and the genomic location. In 2i, high H3K27me3 deposition is scarce with very little enrichment at promoters. In contrast, in serum H3K27me3 is elevated at many genomic locations, 60%–65% of which are promoters (Figures 4A and 4B). In 2i, H3K27me3 is somewhat reduced over long interspersed nuclear element (LINE) repeats (Figure 4C). This is more than offset, however, by much higher levels of H3K27me3 present at satellites. Immunoblotting showed that the total cellular level of H3K27me3 is comparable in 2i and serum (Figure 4D), confirming that the differences at promoters are not secondary to a general reduction in H3K27me3 deposition in 2i.

H3K27me3 is deposited by PRC2 and facilitates recruitment of the PRC1 complex. Transcripts of PRC2 and PRC1 subunits were present at similar levels in 2i and serum (Figure S5A). Transcripts for the H3K27me3 demethylases Kdm6a and

Figure 1. Transcriptome Profiling of ES Cells in 2i and Serum

(A) Fold change (log_2 values) in transcript level of all genes in 2i versus serum. Gene expression values of three ES cells lines derived and maintained in either 2i (TNGA, NOD_male, and NOD_female) or serum (E14, XT67E1, and RGD2) were averaged, after which ratios were calculated. A 2-fold change is indicated by the dotted line. The corresponding heatmap is shown at the bottom. For the remaining analysis, an extra constraint for differential gene expression was set by a p value < 0.2 (Student t test).

(B) RNA-seq levels of a selection of pluripotency, self-renewal, and stem cell markers for 2i and serum ES cells as shown in (A). Expression values for the Rex1-positive serum ES cell population as collected by FACS (Figure S2C) are included.

(C) Functional annotation analysis of the differentially expressed genes between 2i and serum ES cells.

(D) Transcript level of genes associated with the various germ layers.

See also Figure S1, Figure S2, Table S1, Table S2, and Table S3.

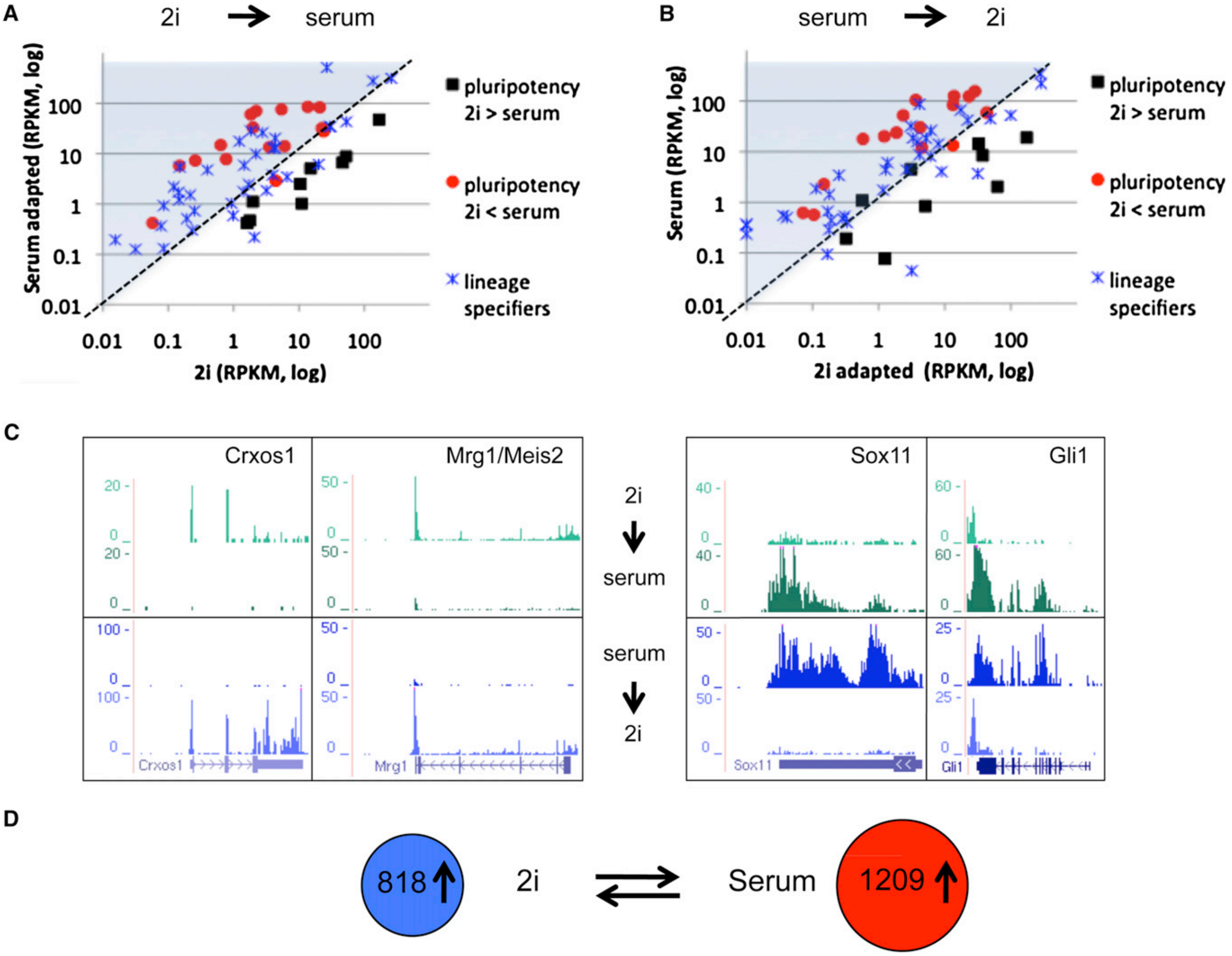

E

Higher 2i

PANTHER biological processes

Term	Count	Total category	P-Value
Other carbon metabolism	11	79	4.9E-05
Other metabolism	32	562	1.3E-04
Lipid, fatty acid and steroid metabolism	38	780	5.8E-04
Fatty acid metabolism	15	192	7.1E-04
Carbohydrate metabolism	28	552	2.1E-03
Coenzyme and prosthetic group metabolism	11	153	8.5E-03
Ion transport	27	601	1.2E-02
Amino acid metabolism	13	219	1.6E-02

KEGG pathway

Term	Count	Total category	P-Value
Glycolysis / Gluconeogenesis	10	68	1.7E-04
Propanoate metabolism	7	30	2.2E-04
Metabolism of xenobiotics by cytochrome P4	9	66	7.2E-04
Lysosome	12	119	8.1E-04

Higher serum

PANTHER biological processes

Term	Count	Total category	P-Value
Developmental processes	176	2106	1.3E-12
Mesoderm development	61	533	2.7E-09
Cell communication	99	1115	1.9E-08
Cell structure and motility	92	1015	2.7E-08
Ectoderm development	67	659	4.6E-08
Neurogenesis	59	579	3.1E-07
Cell adhesion	55	575	5.7E-06
Cell proliferation and differentiation	68	881	3.7E-04
Cell cycle control	29	390	3.4E-02

KEGG pathway

Term	Count	Total category	P-Value
ECM-receptor interaction	19	83	1.8E-07
Pathways in cancer	38	323	4.1E-06
Focal adhesion	27	198	1.1E-05
MAPK signaling pathway	30	265	1.1E-04
TGF-beta signaling pathway	15	87	1.5E-04
Hedgehog signaling pathway	11	54	4.1E-04

Figure 2. Expression Profiles of 2i and Serum ES Cells Are Interconvertible

(A and B) Comparison of expression of pluripotency and lineage-specific genes (as shown in Figures 1B and 1D) for TNGA cells adapted from 2i to serum (A) and E14 adapted from serum to 2i (B).

(C) Typical examples of genes that show transcriptional interconvertibility.

(D) Number of genes that show interconvertibility (>2-fold difference between both 2i and serum conditions).

(E) Functional analysis of the differential genes shown in (D), the genes consistently higher in either 2i or serum.

Kdm6b (also known as Utx and Jmjd3) were also comparable. Ezh2 immunoblotting indicates slightly lower protein in 2i than in serum (Figure S5B). However, phosphorylation of Ezh2 at Thr345, reported to be important for PRC2 recruitment (Kaneko et al., 2010), is similar (Figure S5C). Collectively, these data suggest that the difference in H3K27me3 occupancy at silent promoters in 2i is not primarily attributable to reduced expression of polycomb nor to altered demethylase expression.

Bivalency

Promoters that are marked by H3K27me3 may also display H3K4me3. Such bivalent genes are thought to be poised for activation (Azuara et al., 2006; Bernstein et al., 2006; Mikkelsen et al., 2007). We binned and ranked promoters according to the read density for H3K27me3 (Figure 5A) measured in serum and assessed whether bivalency is preserved in naive ES cells. Applying similar filters and thresholds as used by Mikkelsen et al. (2007), we classified almost 3,000 genes as bivalent in serum (Figure 5B, upper, and Table S5). In 2i, due to the reduced deposition of H3K27me3, many of these genes fall below the threshold, resulting in less than 1,000 genes that qualify as bivalent (Figure 5B, lower). Intensity plots show the general and pronounced diminution in H3K27me3 deposition, whereas H3K4me3 is only slightly altered (Figure 5C). Figure S6A documents the levels of mRNA, H3K27me3, and H3K4me3 in 2i versus serum. Notably, the profiles interconvert upon switching cells between serum and 2i (Figures S6B and S6C).

In both serum and 2i, the bivalent genes are enriched for involvement in developmental processes. Representative examples are the mesoderm specification marker *Hey2* and ectodermal *Metrnl* (Figure 5D). Transcripts are barely detectable in 2i, although H3K4me3 is present and H3K27me3 is low. In serum, transcription is slightly upregulated even though the promoters show a broad gain of H3K27me3. A significant proportion of genes with bivalent promoter marking (31%) exhibit only background transcription in either condition (RPKM < 0.2). However, 14% of the bivalent genes are serum specific (RPKM > 0.5 in serum; RPKM < 0.2 in 2i), whereas a minor fraction (4%) are expressed only in 2i.

In either serum or 2i, H3K27me3 does not colocalize precisely with H3K4me3 but accumulates on either side of the H3K4me3 peak at the transcription start site (Figure 5C). This is consistent with observations that targets of TrxG proteins, which methylate H3K4, show reduced H3K27 methylation (Papp and Müller, 2006; Srinivasan et al., 2008) and that PRC2 activity is inhibited by active marks, including H3K4me3 (Schmitges et al., 2011). Strikingly RNA polymerase II (Pol II) is evident over transcription start sites at higher levels in 2i than in serum (Figure 5C), suggestive of promoter proximal pausing.

Influence of c-Myc

c-Myc is implicated in Pol II pause release (Rahl et al., 2010). We previously noted a very low level of c-Myc protein in 2i (Ying et al., 2008). The RNA-seq data show that *c-myc* mRNA is 40- to 50-fold lower in 2i than in serum and *n-myc* and *l-myc* are also reduced (Figure 1B). We analyzed c-Myc targets that are upregulated in serum (Figure 6A and Figure S7A). Averaged profiles show that promoters of these genes are loaded with H3K4me3 and Pol II in 2i. In serum, Pol II is reduced at the promoters and increased over coding bodies. The Pol II traveling ratio is accordingly increased (Figure 6B), consistent with c-Myc acting as a pause release factor in serum. Typical examples are *Npm1* and *Ncl* (Figure 6C).

We assessed to what extent global differences in transcriptome between 2i and serum might be related to c-Myc. Several differentially expressed genes are c-Myc targets, notably cell-cycle regulators (Figures S7B and S7C). These include cdk/cyclinD components that are positively regulated by Myc and are increased in serum, and conversely cell-cycle inhibitors, p16(Ink4A), p19(Arf), and p21 that are repressed by Myc and upregulated in 2i (Figure S7D). Overall, however, direct c-Myc targets as determined by Chen et al. (2008) represent less than 15% of genes differentially expressed between 2i and serum (Figure 6D). Furthermore, gene ontology classification of c-Myc targets upregulated in serum did not identify categories associated with developmental processes. Therefore Myc is unlikely to be a major determinant of differential expression and metastability.

RNA Polymerase II Pausing

We evaluated the average histone modification profile of all genes that change expression more than 2-fold in 2i versus serum. Genes upregulated in 2i show the expected increased H3K4me3 deposition at the promoter and higher levels of H3K36me3 in the coding body than in serum (Figure 6E and Figure S7E). The repressive mark H3K27me3 is correspondingly reduced. Upregulated genes in serum also show an increase in H3K36me3 over the coding body but in general do not exhibit a significant change in H3K4me3 deposition. More remarkably, on average they show increased H3K27me3.

We then examined Pol II occupancy at these two groups of genes. This showed that upregulation in 2i is reflected in elevated Pol II over the transcriptional start site as well as the coding body (Figure 6F, left). In contrast, genes upregulated in serum showed increased Pol II over the coding body but also on average a reduced signal at the start site (Figure 6F, right). These features indicate that transcriptional elongation at genes already loaded with Pol II is a widespread mechanism of upregulation in serum. RNA-seq data reveal no overt differences in expression of pTEFb components or known regulators of pausing between 2i and serum (Figure S7F). Regulation at the protein level may therefore control differential promoter proximal pause release in naive and metastable ES cells.

Multilineage Differentiation

ES cells maintained in either 2i or serum can colonize the mouse embryo, demonstrating that they are functionally pluripotent. However, they differ markedly in transcription of ectodermal and mesodermal specification genes. The precocious transcription of lineage-associated genes, often termed lineage priming (Hu et al., 1997), is posited to poise stem cells for differentiation. We therefore compared the differentiation behavior of ES cells maintained in 2i or serum. We first used a monolayer neural induction protocol with Sox1GFP reporter ES cells to quantify differentiation (Ying et al., 2003b). Although ES cells maintained

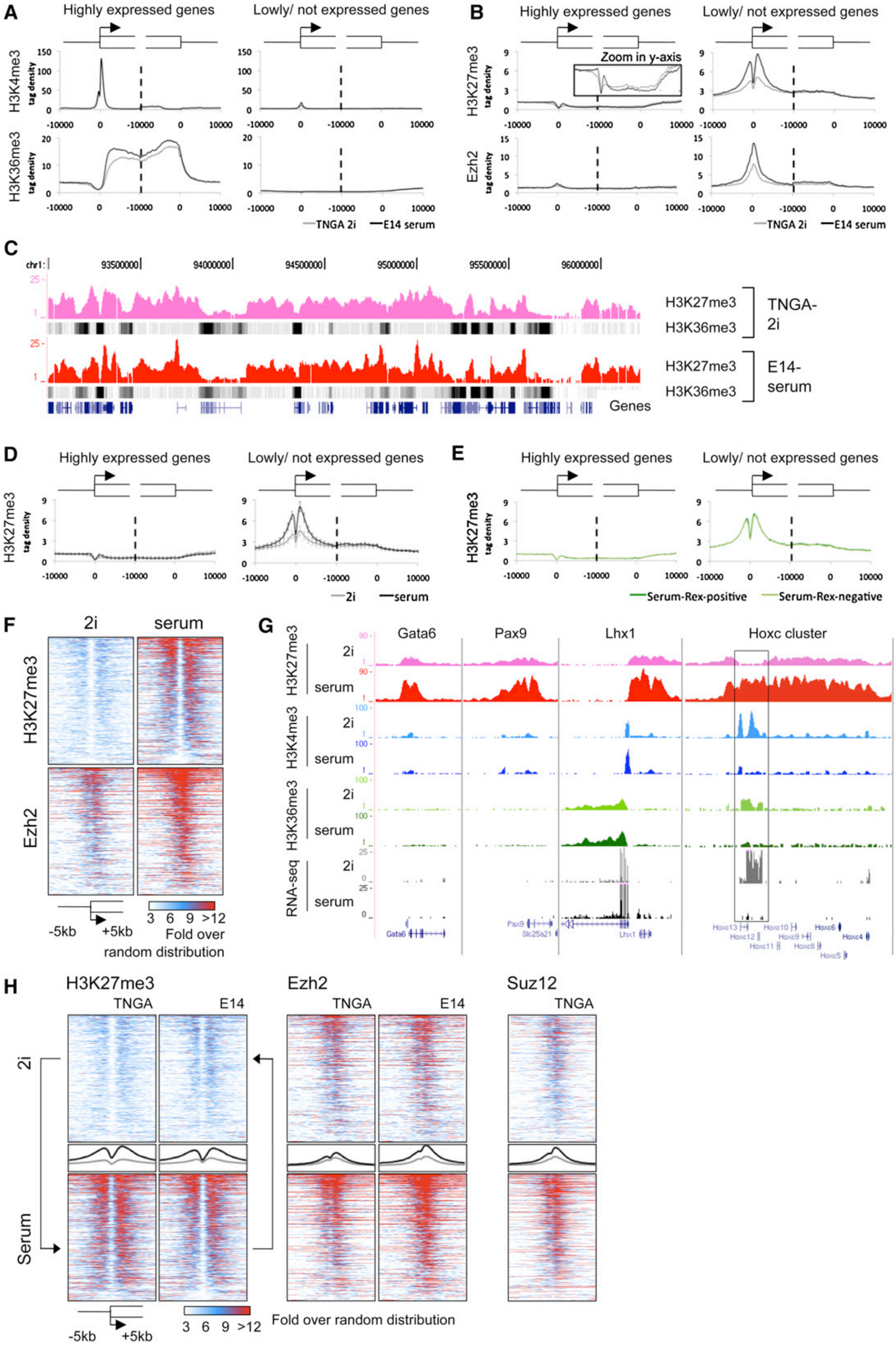

Figure 3. H3K27me3 Is Greatly Diminished at Promoters of Silent Genes and at Hox Clusters in 2i

(A) Average H3K4me3 and H3K36me3 profiles of the 2,000 most active (left plots) and 2,000 silent genes (right plots) from −10kb to +10kb at the transcription start site and the transcription stop. The negative control (genomic DNA) is shown in Figure S3B.

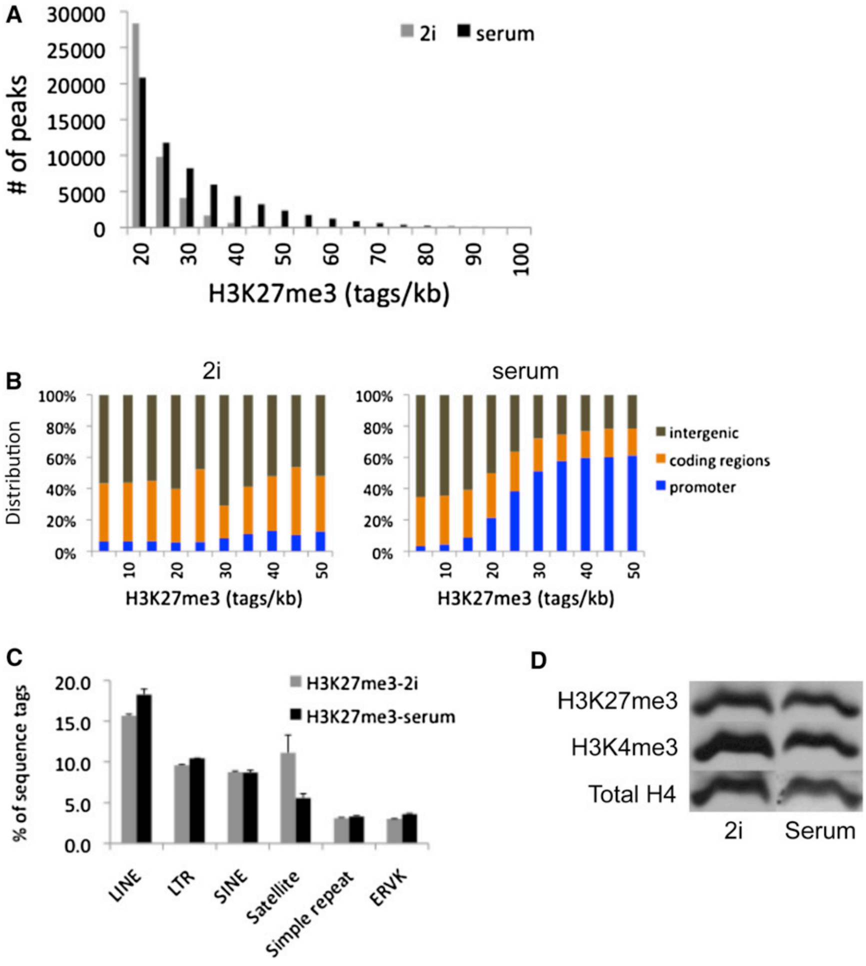

Figure 4. Localization and Quantification of H3K27me3 in 2i and Serum

(A) Binning of H3K27me3 -enriched regions in 2i and serum according to tag densities per peak (in reads/kb). The calculated genomic background is 5 reads/kb per 10 million mapped sequence reads (see Experimental Procedures).

(B) Genomic distribution of H3K27me3 peaks in 2i (left) or serum (right) per bin as shown in (A).

(C) Percentage of H3K27me3 reads present in the major repeat categories.

(D) Immunoblot analysis of histone modifications in total cell extracts.

See also Figure S5.

in serum express several neuroectodermal genes, they were less efficient in generating Sox1GFP-positive neural precursors than ES cells taken from 2i (Figure 7A). This could be due to the presence of cells already biased toward a mesodermal fate in serum. Clearly pre-expression of neural genes in serum does not predispose to this fate. We then used Rex1GFP fractionation to compare ES cell subpopulations in serum with 2i ES cells in embryoid body (EB) differentiation. Rex1GFP-positive cells from serum showed similar behavior to 2i cells (Figure 7B). Downregulation of *Nanog* and *Rex1* was followed by upregulation of the postimplantation epiblast marker *Fgf5*. From 3 days onward, *Fgf5* was downregulated and first *T* (*Brachyury*; although very minor as compared to the Rex1-negative cells), then mesoderm and endoderm lineage markers *Tbx6*, *Cxcr4*, *Sox17*, and *Gata4* appeared. This order is consistent with the developmental progression from blastocyst to gastrulation. In contrast, Rex1GFP-negative cells exhibit accelerated upregulation of *T* and *Tbx6* consistent with their partial differentiation and the loss of self-renewal (Figure S2D).

DISCUSSION

High-resolution genome-wide analyses have revealed that culture environments impose distinctive transcriptional and epigenomic properties on mouse ES cells. In total some 13,000 genes are transcribed at above background levels (>0.2 RPKM). The corollary of this is that around half of genes are effectively inactive. Therefore, undifferentiated ES cells do not show promiscuous gene expression or global transcriptional hyperactivity (Efroni et al., 2008). Nonetheless, the pluripotent transcriptome displays a broad bandwidth; more than 25% of active genes show 2-fold or greater differences between 2i and serum. Around 1,400 genes, predominantly associated with metabolic processes, are upregulated in 2i. In contrast, KEGG analysis points to decreased expression in 2i

(B) Average profiles of H3K27me3 and Ezh2 associated with silencing as in (A). Figure S3G shows a biological replicate analysis for the H3K27me3 profiles of TNGA 2i ES cells.

(C) Typical example of H3K27me3 and H3K36me3 (dense setting) profiles.

(D) H3K27me3 profiling for three different cell lines maintained and derived in either 2i or serum, as in (B). H3K27me3 profiles were generated for TNGA, NOD_male, and NOD_female ES cells in 2i and E14, HM1, and RGD2 ES cells in serum.

(E) As in (B); H3K27me3 profiling in Rex1-positive and Rex1-negative serum ES cells.

(F) H3K27me3 and Ezh2 intensity plots of all promoters containing H3K27me3 at >3-fold over random distribution in at least one of the conditions, TNGA-2i or E14-serum; 3,870 promoters are depicted in rows on the y axis.

(G) Typical examples showing H3K27me3 reduction in 2i as compared to serum.

(H) As in (F); promoter profiles for 2i ES cells adapted to serum and vice versa. Average profiles are plotted in the middle (gray: 2i; black: serum).

See also Figure S3, Figure S4, and Table S4.

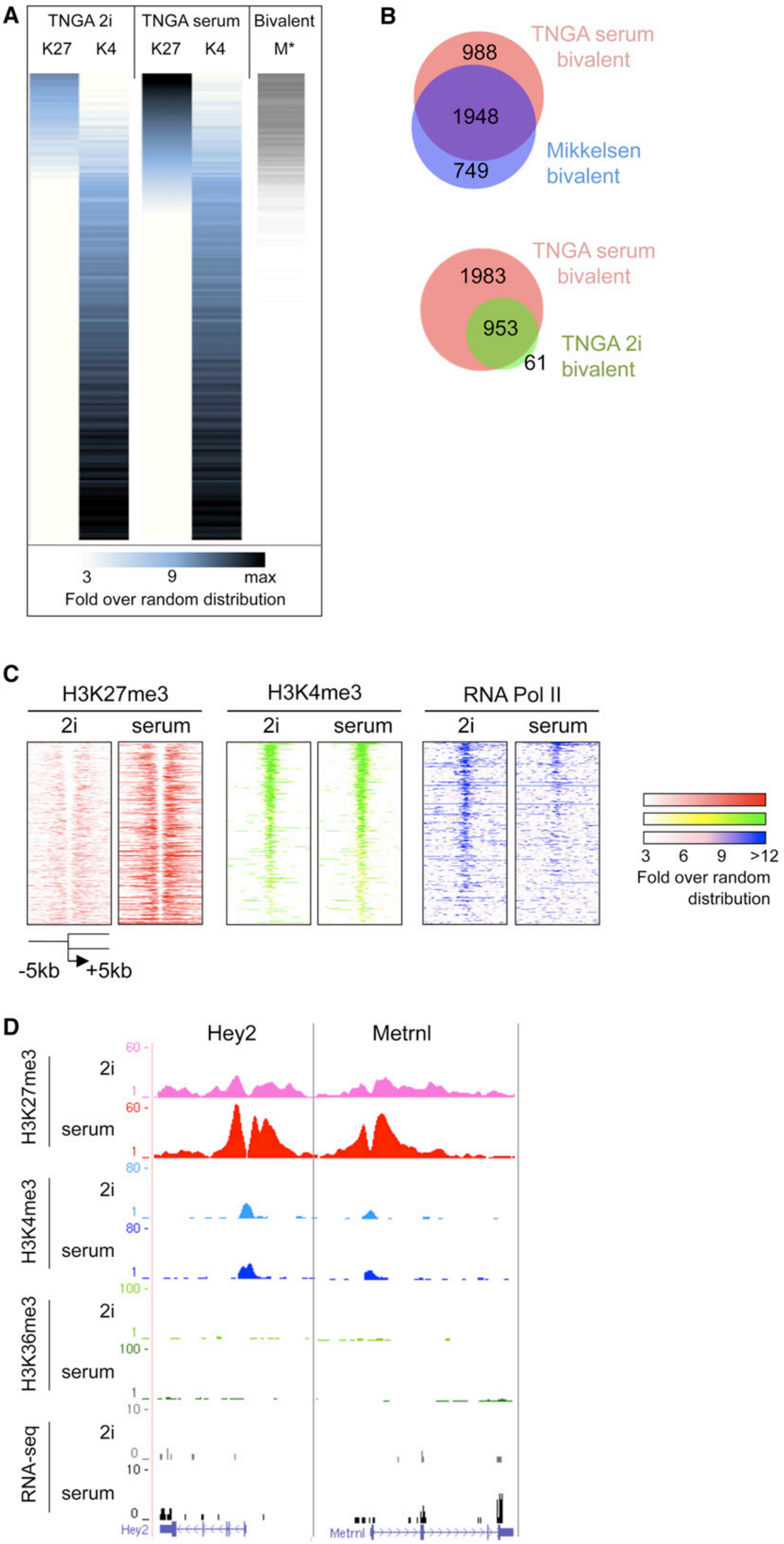

Figure 5. Bivalency in 2i and Serum

(A) H3K27me3 and H3K4me3 intensity plots at the promoters of all genes (sum of reads), ranked on highest to lowest H3K27me3 values in TNGA-serum (blue). Density plot of bivalent genes identified by Mikkelsen et al. (2007; "M") (gray).

(B) Overlap between the bivalent genes as described by Mikkelsen et al. (2007), and the bivalent genes in serum determined in this study (top) and between 2i and serum (bottom).

(C) H3K27me3, H3K4me3, and Pol II intensity plots of all promoters that are bivalent in TNGA serum (this study).

(D) Typical examples of bivalent genes.

See also Figure S6 and Table S5.

of components that might drive differentiation, such as cell communication, mitogen-activated protein kinase (MAPK), and transforming growth factor β (TGFβ and Wnt) pathways. Most strikingly, many ectodermal and mesodermal specification genes that exhibit significant expression in serum are repressed in 2i. Low to absent lineage-affiliated gene expression indicates that multilineage priming is not an intrinsic feature of self-renewing ES cells. Upregulation of such genes in serum suggests that metastability may be an induced condition rather than an inherent property of pluripotent cells.

Some endodermal genes such as *Hex* retain low-level expression in 2i. This may reflect the potential to generate extraembryonic endoderm (Canham et al., 2010). High levels of Prdm14, which has been reported to repress extraembryonic endoderm transcription factors (Ma et al., 2011), may prevent full activation of this program.

Importantly, ES cells transferred between 2i and serum switch their transcriptional profile. Thus a significant component of previously described ES cell signatures reflects an induced serum response. However, critical pluripotency factors are transcribed at similar or only slightly higher levels in 2i. The pluripotency repressors Tcf3 (Wray et al., 2011) and components of the NuRD complex (Kaji et al., 2006) are also expressed at comparable levels. A subset of SCM factors are specifically upregulated in serum, including the *Id* genes that are induced by BMPs or fibronectin and are thought to directly counter the effects of Erk activation (Ying et al., 2003a). Increased Eras, shown to be important for ES cell propagation (Takahashi et al., 2003), and factors such as Sall4, Lin28, and Utf1, may also contribute to reinforcing self-renewal in the face of differentiation stimuli.

The conflict between pluripotency factors and lineage specifiers results in metastability and incipient differentiation in serum. It is suggested that this "precarious balance" (Loh and Lim, 2011) may reflect the circumstance in egg cylinder epiblast cells. However, serum stimulation is an artifactual scenario that may be far from representative of the spatiotemporal precision of

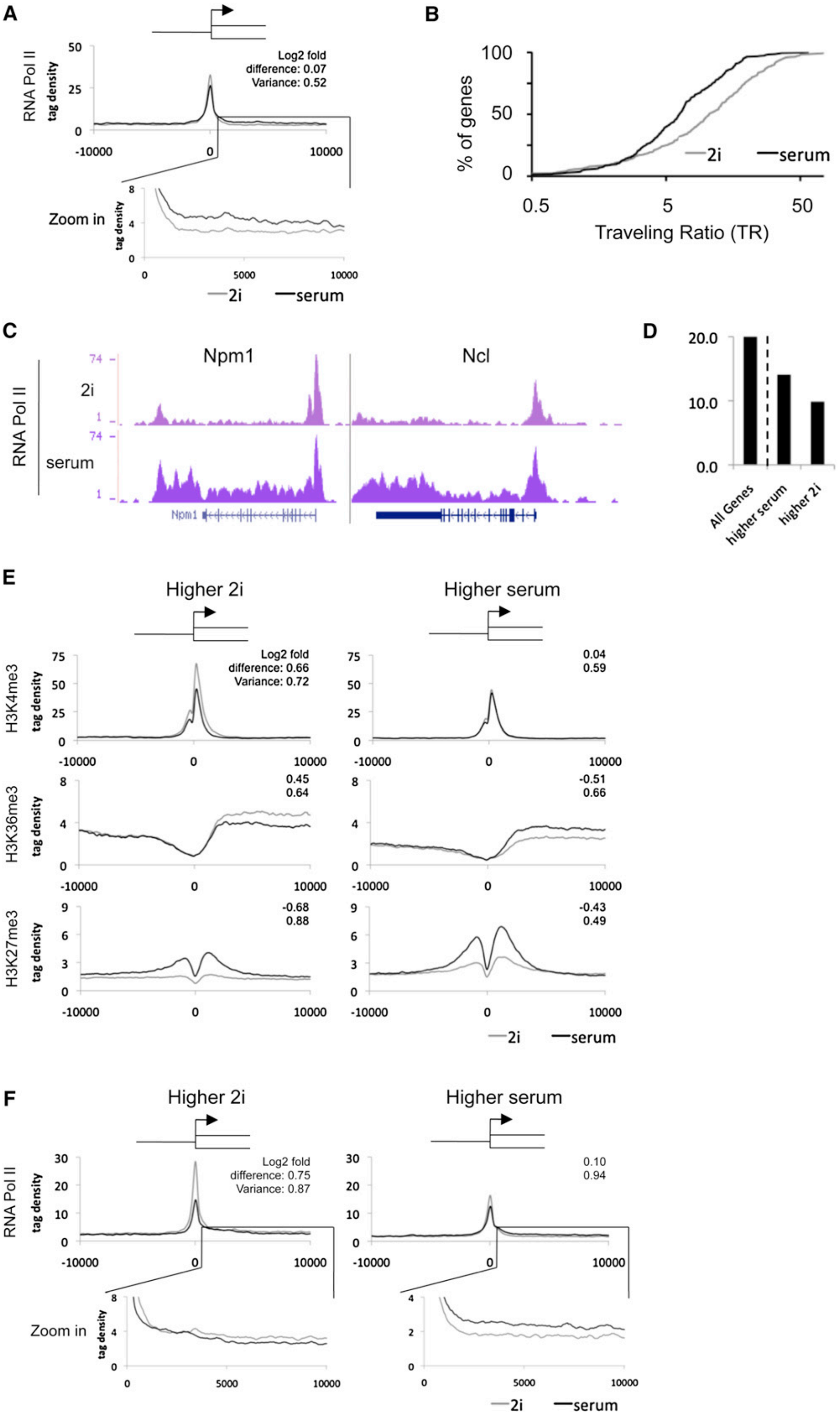
A
RNA Pol II
tag density
Log2 fold
difference: 0.07
Variance: 0.52
Zoom in
2i
serum
B
% of genes
Traveling Ratio (TR)
2i
serum
C
Npm1
Ncl
RNA Pol II
2i
serum
D
All Genes
higher serum
higher 2i
E
Higher 2i
Higher serum
H3K4me3
Log2 fold
difference: 0.66
Variance: 0.72
0.04
0.59
H3K36me3
0.45
0.64
-0.51
0.66
H3K27me3
-0.68
0.88
-0.43
0.49
2i
serum
F
Higher 2i
Higher serum
RNA Pol II
Log2 fold
difference: 0.75
Variance: 0.87
0.10
0.94
Zoom in
2i
serum

inductive stimuli in the embryo. To access postimplantation definitive lineages, ES cells should pass through a phase equivalent to egg cylinder epiblast (Rossant, 2008). Consistent with this, *Fgf5* is upregulated in EBs prior to definitive germ layer markers. From 2i ES cells and the Rex1GFP-positive fraction of serum ES cells, this process follows similar kinetics. Therefore although serum induces transcriptional and epigenetic changes and associated metastability, developmental potential within the Rex1-positive compartment is not fundamentally altered. This is substantiated by the capacity of ES cells from either condition to contribute extensively to chimaeras. However, a significant proportion of cells in serum lose expression of *Rex1* and of core pluripotency factors such as *Nanog* and *Klf4*. They are developmentally more advanced and should be considered distinct from ES cells even though they retain Oct4 (Smith, 2010).

Expression of many genes associated with metabolic and biosynthetic processes is enriched in 2i. This is likely in large part a response to absence of serum constituents, loss of MAPK signaling, and inhibition of GSK3 and indicates that ES cells have adaptable metabolomic capacity. Probably as a consequence of low c-Myc, the cell-cycle inhibitors p16, p19, and p21 are upregulated in 2i, even at the protein level (Figure S7D). Nonetheless, ES cells continue to proliferate rapidly, reflecting their freedom from cyclin checkpoint control (Burdon et al., 2002; Stead et al., 2002). These features can explain the robust expansion of ES cells independent of serum factors and likely underlie their latent tumorigenicity (Chambers and Smith, 2004).

In 2i and serum H3K4me3 peaks are globally similar in number and intensity. In contrast, there is a striking difference in the pattern of H3K27me3 deposition. This mark is present as a lawn across intergenic regions and inactive genes (Figure 3C). However, elevated deposits at promoters of repressed genes are greatly diminished in 2i. The majority of these genes show reduced rather than increased transcription in 2i. This promoter-specific diminution in H3K27me3 is common to multiple ES cell lines. The majority of these genes show reduced rather than increased transcription in 2i. Ezh2 is localized less intensely at promoters in 2i, which may underlie the selective reduction in H3K27me3. Global levels of H3K27me3 are similar in 2i and serum. Indeed, H3K27me3 is increased at satellites in 2i, indicating that these may serve as a sink. Notably, there is no change in H3K9me3 over satellites (Figure S3C).

In 2i only around 1,000 genes have both H3K4me3 and H3K27me3 marks, which argues against bivalency as a master epigenetic blueprint. Nonetheless, most of the remaining bivalent genes can be classified as developmental. In serum, more genes are bivalent due to acquisition of H3K27me3. Surprisingly, this is accompanied by a slight overall increase in expression, although the majority remain silent or transcribed at very low levels (Figure S6A). It is conceivable that although the local levels of PRC2 and H3K27me3 are reduced in 2i, they remain sufficient to repress transcription. It should be noted, however, that ES cells lacking PRC2, PRC1, or both are viable and show derepression of lineage-specific markers to only a low level (Leeb et al., 2010). Our findings are thus in line with genetic evidence that polycomb is not a central mechanism for silencing gene expression in the naive state and only becomes critical during differentiation.

RNA polymerase pausing has been identified by GRO-seq analysis (Min et al., 2011) at variable extents at many genes in ES cells cultured in serum. Our findings indicate that pausing is more prevalent in 2i than serum. Induction of c-Myc in serum may facilitate pause release at some loci. This is consistent with recent evidence that Myc function is unnecessary in naive ES cells but required in serum (Hishida et al., 2011). However, many of the genes whose expression is most markedly upregulated in serum, including germ layer specification factors, are not reported Myc targets. Therefore additional mechanisms are likely to control pause release in ES cells.

In mammals pluripotent cells harbor the germline and most pluripotency factors are also key players in germ cell specification and differentiation. It is interesting therefore that in *Caenorhabditis elegans* and *Drosophila*, germline development is dependent on transcriptional pausing mediated at the level of pTEFb antagonism by Pie-1 and Pgc, respectively (Nakamura and Seydoux, 2008). This raises the question of whether naive ES cells might contain an analogous factor that interferes with pTEFb to suppress transcriptional elongation. It will also be revealing to determine whether Erk signaling may cause activation of pTEFb (Fujita et al., 2008; Lee et al., 2010).

Recruitment and pausing of RNA polymerase II with lack of consolidated H3K27me3 silencing may constitute a potentiated template for induction of lineage-specific transcription programs. Pausing may serve to minimize the effects of noise and ensure rapid, coordinated, and synchronous gene induction in response to developmental cues or extrinsic stimuli (Boettiger and Levine, 2009; Nechaev and Adelman, 2011). Recent studies also indicate that Pol II pausing inhibits nucleosome assembly (Gilchrist et al., 2010) and could thereby influence histone modification profile. Interestingly, in *Xenopus* embryos H3K27me3 is not deposited during zygotic gene activation but is acquired later and associated with spatial restriction of gene expression (Akkers et al., 2009). In the mouse ICM, H3K27me localization has not been determined, but various epigenetic silencing

Figure 6. RNA Polymerase II Pausing in Naive ES Cells

(A) Averaged Pol II at promoters of Myc-targets upregulated in serum. The top corner values represent the average $\log_2$-fold difference of the individual data points, and the variance, between 2i and serum.

(B) Pol II traveling ratio (a quantification of pause release) of the Myc targets upregulated in serum.

(C) Typical examples of two Myc target genes showing pause release of Pol II.

(D) Percentage of Myc targets among all genes (left) or among the genes higher (>2-fold change) in serum or 2i (right).

(E) Averaged profiles of the promoter region of genes that are more highly expressed in 2i (left) or serum (right).

(F) Averaged Pol II profiles for the same gene groups as in (E).

See also Figure S7.

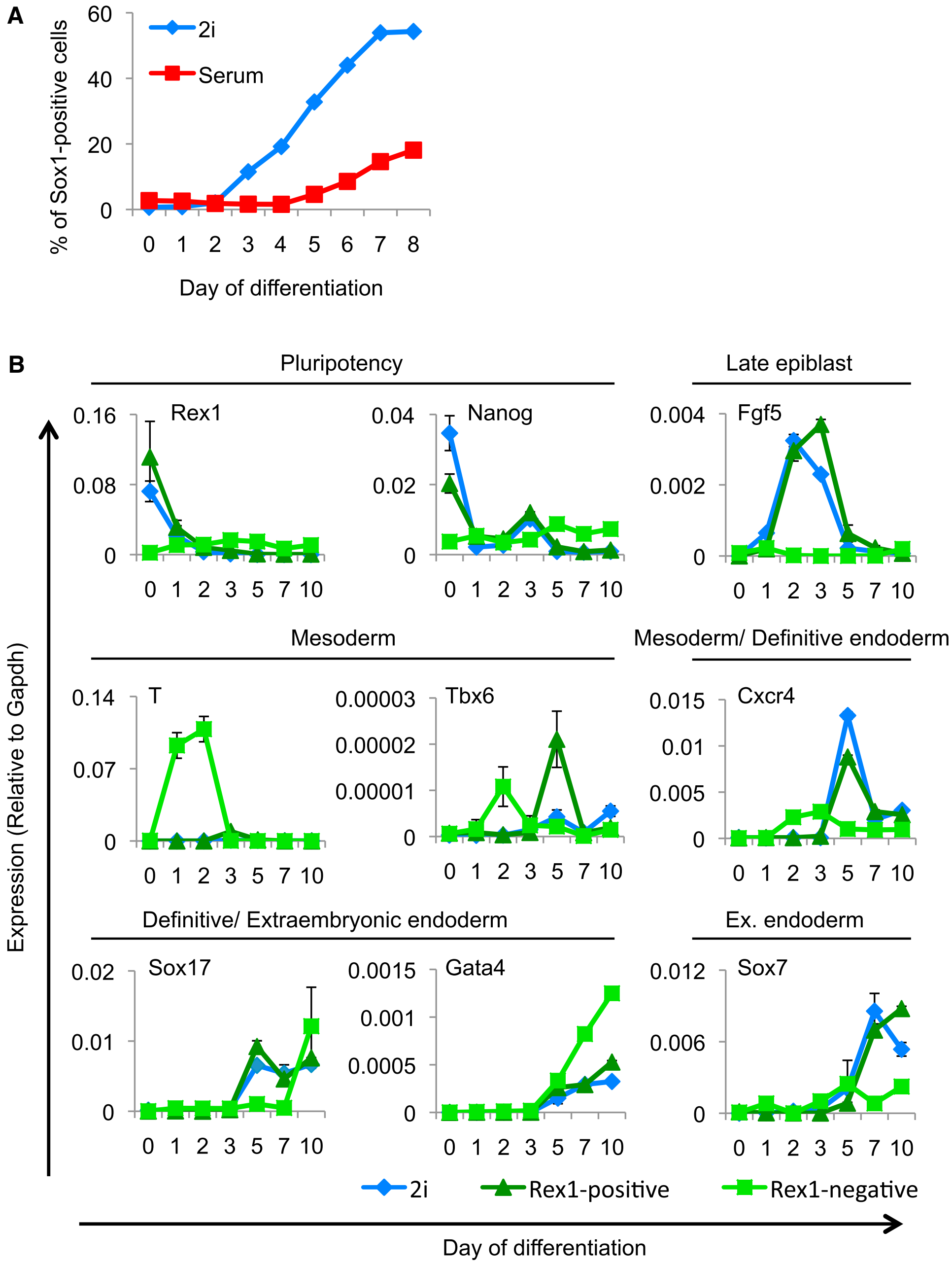

Figure 7. Differentiation Kinetics/Potential of 2i and Serum ES Cells

(A) Monolayer neural differentiation of Sox1-GFP ES cells maintained in either 2i or serum.

(B) Embryoid body differentiation of cells maintained in 2i, and of Rex1-positive and Rex1-negative serum ES cells as sorted by FACS (Figure S2C). Expression levels were determined by RT-qPCR.

components appear to be expressed at low levels (Tang et al., 2010).

Collectively the observations reported here yield insights into the molecular underpinning of naive pluripotency and revise previous assumptions derived from analysis of heterogeneous and metastable serum-treated cultures. The findings provoke questions about the regulation of gene expression in pluripotent cells and the process of lineage specification. Transcriptional potentiation through promoter proximal pausing may play a major role in the establishment and stable maintenance of naive pluripotency. Currently there is great interest in isolating human pluripotent stem cells in a naive state (Hanna et al., 2010; Wang et al., 2011). The distinctive transcriptome and epigenome characteristics of ground state mouse ES cells may provide a valuable criterion against which to measure such claims. In addition, these data sets provide a benchmark

resource for analysis and modeling of gene expression control during self-renewal and in the transition from naive pluripotency to lineage commitment.

EXPERIMENTAL PROCEDURES

Cell Culture and Methods

ES cells were cultured without feeders in the presence of leukemia inhibitory factor (LIF) either in Glasgow modification of Eagles medium (GMEM) containing 10% fetal calf serum or in serum-free N2B27 supplemented with MEK inhibitor PD0325901 (1 μM) and GSK3 inhibitor CH99021 (3 μM), together known as 2i (Ying et al., 2008). E14Tg2a (E14), Rex1GFPd2 (RGD2, Rex1GFP), and HM1 are male ES cells of 129 background established and maintained in serum without feeders. The serum-derived female XT67E1 line is from a mixed 129 and PGK/C3H background (Penny et al., 1996). TNGA female ES cells were derived and maintained in 2i from embryos on a mixed strain 129 and C57BL/6 background heterozygous for eGFP knock-in at the *Nanog* gene (Chambers et al., 2007). Female and male ES cells from the nonobese diabetic (NOD) strain were derived and maintained in 2i (Nichols et al., 2009). Chromatin immunoprecipitations (ChIP) were performed as described (Marks et al., 2009). Cell sorting (fluorescence-activated cell sorting [FACS]), immunoblotting, RNA isolation, and cDNA synthesis were performed according to standard protocols described in the Extended Experimental Procedures, which also lists antibodies used.

Sequencing

DNA samples were prepared for sequencing by end repair of 20 ng DNA as measured by Qubit (Invitrogen). Adaptors were ligated to DNA fragments, followed by size selection (~300 bp) and 14 cycles of PCR amplification. Cluster generation and sequencing (36 bp) was performed with the Illumina Genome Analyzer IIx (GAIIx) platform according to standard Illumina protocols. The standard pipeline to generate the sequencing output files are described in the Supplemental Information. All sequencing analyses were conducted based on the *Mus musculus* NCBI m37 genome assembly (MM9; assembly July 2007). Table S1 summarizes the sequencing output. All RNA-seq and ChIP-seq data (FASTQ, BED, and WIG files) are present in the NCBI GEO SuperSeries GSE23943.

RNA-Seq Analysis

To obtain RNA-Seq gene expression values (RPKM), we used Genomatix (www.genomatix.de). Differential genes were called at a 2-fold difference and $p < 0.2$ in a Student t test (among three biological replicates for both 2i and serum). The identification of ncRNAs is described in the Extended Experimental Procedures. GO and KEGG analysis was performed with DAVID (http://david.abcc.ncifcrf.gov/).

ChIP-Seq Analyses

To compensate for differences in sequencing depth and mapping efficiency, the total number of unique reads of each sample was uniformly equalized, allowing quantitative comparisons. Tag densities on the average profiles were determined by calculating tag density over each base pair (using a 40 bp window size) per 10 million total mapped reads. The genes used for the average epigenetic profiles were based on the 2,000 most active/inactive genes in TNGA-2i. The 2,000 lowest/not expressed genes were selected by the additional requirement of H3K27me3 promoter enrichments of > 3-fold over background in either TNGA-2i or E14-serum. Random distribution values were determined by calculating average read densities of the genomic DNA profile (4.962 reads/kb at 10 million sequenced reads equivalent to an average density of 1.489 per bp). Genes were considered bivalent if both H3K4me3 and H3K27me3 were > 3-fold over random distribution, similar to criteria applied by Mikkelsen et al. (2007). The RNA Polymerase II traveling was calculated as described by Rahl et al. (2010). The Chip-seq repeat analysis procedure is described in the Supplemental Information.

Differentiation Assays

For monolayer neural differentiation, we used Sox1-GFP (46C) ES cells, which contain a GFP knock-in at the endogenous Sox1 locus (Ying et al., 2003b). Cells cultured in 2i or serum were plated at a density of 5,000 cells per cm^2. Sixteen hours after plating, media were switched to N2B27 to induce neural differentiation. Percentage of GFP-positive cells was determined by flow cytometry. For EB differentiation, single EBs were formed by sorting 1,500 cells into each well of PrimeSurface96U plates containing 15% serum and no LIF. Sixteen EBs were pooled each day and analyzed by RT-qPCR with TaqMan probes (Applied Biosystems).

ACCESSION NUMBERS

The GEO accession number for the SuperSeries reported in this paper is GSE23943.

SUPPLEMENTAL INFORMATION

Supplemental Information includes Supplemental Experimental Procedures, seven figures, and six tables and can be found with this article online at doi:10.1016/j.cell.2012.03.026.

ACKNOWLEDGMENTS

We thank Eva Janssen-Megens, Anita Kaan, and Yan Tan for sequencing; Kees-Jan Françoijs, Hinri Kerstens, Simon van Heeringen, and Arjen Brinkman for bioinformatic assistance; and Rachel Walker for help with flow cytometry. We thank Paul Bertone for comments on the manuscript. Tatyana Nesterova and Neil Brockdorff provided the XT67E1 cells and Olga Ujhelly and Andras Dinnyes the HM1 cells. The pThr345-Ezh2 antibody was a gift from Danny Reinberg. The research leading to these results has received funding from the European Union grants HEROIC (18883; FP6/2005-2010), PluriSys (223485; FP7/2009), EuroSyStem (200720; FP7/2008), and ATLAS (221952; FP7/2009), the Deutsche Forschungsgemeinschaft SPP1356 Pluripotency and Reprogramming, and The Wellcome Trust. A.S. is a Medical Research Council Professor.

Received: July 9, 2011
Revised: December 26, 2011
Accepted: March 6, 2012
Published: April 26, 2012

REFERENCES

Akkers, R.C., van Heeringen, S.J., Jacobi, U.G., Janssen-Megens, E.M., Françoijs, K.J., Stunnenberg, H.G., and Veenstra, G.J. (2009). A hierarchy of H3K4me3 and H3K27me3 acquisition in spatial gene regulation in Xenopus embryos. Dev. Cell *17*, 425–434.

Azuara, V., Perry, P., Sauer, S., Spivakov, M., Jørgensen, H.F., John, R.M., Gouti, M., Casanova, M., Warnes, G., Merkenschlager, M., and Fisher, A.G. (2006). Chromatin signatures of pluripotent cell lines. Nat. Cell Biol. *8*, 532–538.

Bernstein, B.E., Mikkelsen, T.S., Xie, X., Kamal, M., Huebert, D.J., Cuff, J., Fry, B., Meissner, A., Wernig, M., Plath, K., et al. (2006). A bivalent chromatin structure marks key developmental genes in embryonic stem cells. Cell *125*, 315–326.

Boettiger, A.N., and Levine, M. (2009). Synchronous and stochastic patterns of gene activation in the Drosophila embryo. Science *325*, 471–473.

Buehr, M., Meek, S., Blair, K., Yang, J., Ure, J., Silva, J., McLay, R., Hall, J., Ying, Q.L., and Smith, A. (2008). Capture of authentic embryonic stem cells from rat blastocysts. Cell *135*, 1287–1298.

Burdon, T., Smith, A., and Savatier, P. (2002). Signalling, cell cycle and pluripotency in embryonic stem cells. Trends Cell Biol. *12*, 432–438.

Canham, M.A., Sharov, A.A., Ko, M.S., and Brickman, J.M. (2010). Functional heterogeneity of embryonic stem cells revealed through translational amplification of an early endodermal transcript. PLoS Biol. *8*, e1000379.

Cao, R., Wang, L., Wang, H., Xia, L., Erdjument-Bromage, H., Tempst, P., Jones, R.S., and Zhang, Y. (2002). Role of histone H3 lysine 27 methylation in Polycomb-group silencing. Science *298*, 1039–1043.

Chambers, I., and Smith, A. (2004). Self-renewal of teratocarcinoma and embryonic stem cells. Oncogene *23*, 7150–7160.

Chambers, I., Silva, J., Colby, D., Nichols, J., Nijmeijer, B., Robertson, M., Vrana, J., Jones, K., Grotewold, L., and Smith, A. (2007). Nanog safeguards pluripotency and mediates germline development. Nature *450*, 1230–1234.

Chen, X., Xu, H., Yuan, P., Fang, F., Huss, M., Vega, V.B., Wong, E., Orlov, Y.L., Zhang, W., Jiang, J., et al. (2008). Integration of external signaling pathways with the core transcriptional network in embryonic stem cells. Cell *133*, 1106–1117.

Efroni, S., Duttagupta, R., Cheng, J., Dehghani, H., Hoeppner, D.J., Dash, C., Bazett-Jones, D.P., Le Grice, S., McKay, R.D., Buetow, K.H., et al. (2008). Global transcription in pluripotent embryonic stem cells. Cell Stem Cell *2*, 437–447.

Fujita, T., Ryser, S., Piuz, I., and Schlegel, W. (2008). Up-regulation of P-TEFb by the MEK1-extracellular signal-regulated kinase signaling pathway contributes to stimulated transcription elongation of immediate early genes in neuroendocrine cells. Mol. Cell. Biol. *28*, 1630–1643.

Gilchrist, D.A., Dos Santos, G., Fargo, D.C., Xie, B., Gao, Y., Li, L., and Adelman, K. (2010). Pausing of RNA polymerase II disrupts DNA-specified nucleosome organization to enable precise gene regulation. Cell *143*, 540–551.

Graf, T., and Stadtfeld, M. (2008). Heterogeneity of embryonic and adult stem cells. Cell Stem Cell *3*, 480–483.

Guo, G., Huss, M., Tong, G.Q., Wang, C., Li Sun, L., Clarke, N.D., and Robson, P. (2010). Resolution of cell fate decisions revealed by single-cell gene expression analysis from zygote to blastocyst. Dev. Cell *18*, 675–685.

Guttman, M., Garber, M., Levin, J.Z., Donaghey, J., Robinson, J., Adiconis, X., Fan, L., Koziol, M.J., Gnirke, A., Nusbaum, C., et al. (2010). Ab initio reconstruction of cell type-specific transcriptomes in mouse reveals the conserved multi-exonic structure of lincRNAs. Nat. Biotechnol. *28*, 503–510.

Hanna, J., Markoulaki, S., Mitalipova, M., Cheng, A.W., Cassady, J.P., Staerk, J., Carey, B.W., Lengner, C.J., Foreman, R., Love, J., et al. (2009). Metastable pluripotent states in NOD-mouse-derived ESCs. Cell Stem Cell *4*, 513–524.

Hanna, J., Cheng, A.W., Saha, K., Kim, J., Lengner, C.J., Soldner, F., Cassady, J.P., Muffat, J., Carey, B.W., and Jaenisch, R. (2010). Human embryonic stem cells with biological and epigenetic characteristics similar to those of mouse ESCs. Proc. Natl. Acad. Sci. USA *107*, 9222–9227.

Hayashi, K., Lopes, S.M., Tang, F., and Surani, M.A. (2008). Dynamic equilibrium and heterogeneity of mouse pluripotent stem cells with distinct functional and epigenetic states. Cell Stem Cell *3*, 391–401.

Hishida, T., Nozaki, Y., Nakachi, Y., Mizuno, Y., Okazaki, Y., Ema, M., Takahashi, S., Nishimoto, M., and Okuda, A. (2011). Indefinite self-renewal of ESCs through Myc/Max transcriptional complex-independent mechanisms. Cell Stem Cell *9*, 37–49.

Hu, M., Krause, D., Greaves, M., Sharkis, S., Dexter, M., Heyworth, C., and Enver, T. (1997). Multilineage gene expression precedes commitment in the hemopoietic system. Genes Dev. *11*, 774–785.

Ivanova, N., Dobrin, R., Lu, R., Kotenko, I., Levorse, J., DeCoste, C., Schafer, X., Lun, Y., and Lemischka, I.R. (2006). Dissecting self-renewal in stem cells with RNA interference. Nature *442*, 533–538.

Kaji, K., Caballero, I.M., MacLeod, R., Nichols, J., Wilson, V.A., and Hendrich, B. (2006). The NuRD component Mbd3 is required for pluripotency of embryonic stem cells. Nat. Cell Biol. *8*, 285–292.

Kalmar, T., Lim, C., Hayward, P., Muñoz-Descalzo, S., Nichols, J., Garcia-Ojalvo, J., and Martinez Arias, A. (2009). Regulated fluctuations in nanog expression mediate cell fate decisions in embryonic stem cells. PLoS Biol. *7*, e1000149.

Kaneko, S., Li, G., Son, J., Xu, C.F., Margueron, R., Neubert, T.A., and Reinberg, D. (2010). Phosphorylation of the PRC2 component Ezh2 is cell cycle-regulated and up-regulates its binding to ncRNA. Genes Dev. *24*, 2615–2620.

Kiyonari, H., Kaneko, M., Abe, S., and Aizawa, S. (2010). Three inhibitors of FGF receptor, ERK, and GSK3 establishes germline-competent embryonic stem cells of C57BL/6N mouse strain with high efficiency and stability. Genesis *48*, 317–327.

Kunath, T., Saba-El-Leil, M.K., Almousailleakh, M., Wray, J., Meloche, S., and Smith, A. (2007). FGF stimulation of the Erk1/2 signalling cascade triggers transition of pluripotent embryonic stem cells from self-renewal to lineage commitment. Development *134*, 2895–2902.

Lee, J.S., Smith, E., and Shilatifard, A. (2010). The language of histone crosstalk. Cell *142*, 682–685.

Leeb, M., Pasini, D., Novatchkova, M., Jaritz, M., Helin, K., and Wutz, A. (2010). Polycomb complexes act redundantly to repress genomic repeats and genes. Genes Dev. *24*, 265–276.

Li, P., Tong, C., Mehrian-Shai, R., Jia, L., Wu, N., Yan, Y., Maxson, R.E., Schulze, E.N., Song, H., Hsieh, C.L., et al. (2008). Germline competent embryonic stem cells derived from rat blastocysts. Cell *135*, 1299–1310.

Loh, K.M., and Lim, B. (2011). A precarious balance: pluripotency factors as lineage specifiers. Cell Stem Cell *8*, 363–369.

Ma, Z., Swigut, T., Valouev, A., Rada-Iglesias, A., and Wysocka, J. (2011). Sequence-specific regulator Prdm14 safeguards mouse ESCs from entering extraembryonic endoderm fates. Nat. Struct. Mol. Biol. *18*, 120–127.

Marks, H., Chow, J.C., Denissov, S., Françoijs, K.J., Brockdorff, N., Heard, E., and Stunnenberg, H.G. (2009). High-resolution analysis of epigenetic changes associated with X inactivation. Genome Res. *19*, 1361–1373.

Mikkelsen, T.S., Ku, M., Jaffe, D.B., Issac, B., Lieberman, E., Giannoukos, G., Alvarez, P., Brockman, W., Kim, T.K., Koche, R.P., et al. (2007). Genome-wide maps of chromatin state in pluripotent and lineage-committed cells. Nature *448*, 553–560.

Min, I.M., Waterfall, J.J., Core, L.J., Munroe, R.J., Schimenti, J., and Lis, J.T. (2011). Regulating RNA polymerase pausing and transcription elongation in embryonic stem cells. Genes Dev. *25*, 742–754.

Nakamura, A., and Seydoux, G. (2008). Less is more: specification of the germline by transcriptional repression. Development *135*, 3817–3827.

Nechaev, S., and Adelman, K. (2011). Pol II waiting in the starting gates: Regulating the transition from transcription initiation into productive elongation. Biochim. Biophys. Acta *1809*, 34–45.

Nichols, J., Jones, K., Phillips, J.M., Newland, S.A., Roode, M., Mansfield, W., Smith, A., and Cooke, A. (2009). Validated germline-competent embryonic stem cell lines from nonobese diabetic mice. Nat. Med. *15*, 814–818.

Niwa, H., Ogawa, K., Shimosato, D., and Adachi, K. (2009). A parallel circuit of LIF signalling pathways maintains pluripotency of mouse ES cells. Nature *460*, 118–122.

Papp, B., and Müller, J. (2006). Histone trimethylation and the maintenance of transcriptional ON and OFF states by trxG and PcG proteins. Genes Dev. *20*, 2041–2054.

Penny, G.D., Kay, G.F., Sheardown, S.A., Rastan, S., and Brockdorff, N. (1996). Requirement for Xist in X chromosome inactivation. Nature *379*, 131–137.

Rahl, P.B., Lin, C.Y., Seila, A.C., Flynn, R.A., McCuine, S., Burge, C.B., Sharp, P.A., and Young, R.A. (2010). c-Myc regulates transcriptional pause release. Cell *141*, 432–445.

Rinn, J.L., Kertesz, M., Wang, J.K., Squazzo, S.L., Xu, X., Brugmann, S.A., Goodnough, L.H., Helms, J.A., Farnham, P.J., Segal, E., and Chang, H.Y. (2007). Functional demarcation of active and silent chromatin domains in human HOX loci by noncoding RNAs. Cell *129*, 1311–1323.

Rossant, J. (2008). Stem cells and early lineage development. Cell *132*, 527–531.

Schmitges, F.W., Prusty, A.B., Faty, M., Stützer, A., Lingaraju, G.M., Aiwazian, J., Sack, R., Hess, D., Li, L., Zhou, S., et al. (2011). Histone methylation by PRC2 is inhibited by active chromatin marks. Mol. Cell *42*, 330–341.

Smith, A.G. (2001). Embryo-derived stem cells: of mice and men. Annu. Rev. Cell Dev. Biol. *17*, 435–462.

Smith, A. (2010). Pluripotent stem cells: private obsession and public expectation. EMBO Mol. Med. *2*, 113–116.

Srinivasan, S., Dorighi, K.M., and Tamkun, J.W. (2008). Drosophila Kismet regulates histone H3 lysine 27 methylation and early elongation by RNA polymerase II. PLoS Genet. *4*, e1000217.

Stead, E., White, J., Faast, R., Conn, S., Goldstone, S., Rathjen, J., Dhingra, U., Rathjen, P., Walker, D., and Dalton, S. (2002). Pluripotent cell division cycles are driven by ectopic Cdk2, cyclin A/E and E2F activities. Oncogene *21*, 8320–8333.

Stock, J.K., Giadrossi, S., Casanova, M., Brookes, E., Vidal, M., Koseki, H., Brockdorff, N., Fisher, A.G., and Pombo, A. (2007). Ring1-mediated ubiquitination of H2A restrains poised RNA polymerase II at bivalent genes in mouse ES cells. Nat. Cell Biol. *9*, 1428–1435.

Takahashi, K., Mitsui, K., and Yamanaka, S. (2003). Role of ERas in promoting tumour-like properties in mouse embryonic stem cells. Nature *423*, 541–545.

Tang, F., Barbacioru, C., Bao, S., Lee, C., Nordman, E., Wang, X., Lao, K., and Surani, M.A. (2010). Tracing the derivation of embryonic stem cells from the inner cell mass by single-cell RNA-Seq analysis. Cell Stem Cell *6*, 468–478.

Toyooka, Y., Shimosato, D., Murakami, K., Takahashi, K., and Niwa, H. (2008). Identification and characterization of subpopulations in undifferentiated ES cell culture. Development *135*, 909–918.

Wang, W., Yang, J., Liu, H., Lu, D., Chen, X., Zenonos, Z., Campos, L.S., Rad, R., Guo, G., Zhang, S., et al. (2011). Rapid and efficient reprogramming of somatic cells to induced pluripotent stem cells by retinoic acid receptor gamma and liver receptor homolog 1. Proc. Natl. Acad. Sci. USA *108*, 18283–18288.

Wray, J., Kalkan, T., and Smith, A.G. (2010). The ground state of pluripotency. Biochem. Soc. Trans. *38*, 1027–1032.

Wray, J., Kalkan, T., Gomez-Lopez, S., Eckardt, D., Cook, A., Kemler, R., and Smith, A. (2011). Inhibition of glycogen synthase kinase-3 alleviates Tcf3 repression of the pluripotency network and increases embryonic stem cell resistance to differentiation. Nat. Cell Biol. *13*, 838–845.

Ying, Q.L., Nichols, J., Chambers, I., and Smith, A. (2003a). BMP induction of Id proteins suppresses differentiation and sustains embryonic stem cell self-renewal in collaboration with STAT3. Cell *115*, 281–292.

Ying, Q.L., Stavridis, M., Griffiths, D., Li, M., and Smith, A. (2003b). Conversion of embryonic stem cells into neuroectodermal precursors in adherent monoculture. Nat. Biotechnol. *21*, 183–186.

Ying, Q.L., Wray, J., Nichols, J., Batlle-Morera, L., Doble, B., Woodgett, J., Cohen, P., and Smith, A. (2008). The ground state of embryonic stem cell self-renewal. Nature *453*, 519–523.

Young, R.A. (2011). Control of the embryonic stem cell state. Cell *144*, 940–954.

EXTENDED EXPERIMENTAL PROCEDURES

Double-Stranded cDNA Synthesis

Total RNA was isolated with Trizol (Invitrogen) according to the manufacturer's recommendations. 100 μg total RNA was subjected to two rounds of poly(A) selection (Oligotex mRNA Mini Kit; QIAGEN), followed by DNaseI treatment (QIAGEN). 100–200 ng mRNA was fragmented by hydrolysis (5× fragmentation buffer: 200mM Tris acetate, pH8.2, 500mM potassium acetate and 150mM magnesium acetate) at 94°C for 90 s and purified (RNAeasy Minelute Kit; QIAGEN). cDNA was synthesized with 5 μg random hexamers by Superscript III Reverse Transcriptase (Invitrogen). Ds cDNA synthesis was performed in second strand buffer (Invitrogen) according to the manufacturer's recommendations and purified (Minelute Reaction Cleanup Kit; QIAGEN). Strand-specific rRNA depleted ds cDNA profiling was performed with the ScriptSeq kit (cat. no. SS10924) from Illumina, according to the instructions of the manufacturer. rRNA depletion was performed with the Ribo-Zero rRNA Removal Kit (Human/Mouse/Rat; cat. no. RZH110424). Validation experiments were performed by RT-qPCR with primers as shown in Table S6.

ChIP

Experiments were performed with 3.3×10^6 cells and 3 μg antibody per ChIP as described (Marks et al., 2009) with two minor modifications. Crosslinking was performed on the culture plates for 20 min and ChIP'ed DNA was purified by Qiaquick PCR purification Kit (QIAGEN). ChIP enrichment levels were analyzed by qPCR for quality control. Antibodies used for ChIP are described in the supplemental information. Validation experiments were performed by qPCR with primers as shown in Table S6.

Antibodies Used for ChIP

The following polyclonal antibodies were used for ChIP: H3K4me3 (Diagenode pAb-MEHAHS-024, A1-010); H3K27me3 (Millipore 07-449, DAM-1588246); H3K36me3 (Diagenode CS-058-100, A114-001); H3K9me3 (Abcam ab8898-100, lot 733953) Ezh2 (Active Motif 39639, 23809001); RNA Polymerase II (Diagenode AC-055-100, 001; also known as the 8wg16 RNA Polymerase II antibody, with the nonphosphorylated Ser2 of the RNA Polymerase II carboxyl-terminal domain (CTD) consensus sequence repeat YSPTSPS as main target. This antibody recognizes unphosphorylated (initiating) and Ser5 only phosphorylated RNA Polymerase II (Morris et al., 2005)).

Immunoblot Analysis

For immunoblot analysis, 3 μg of histone extracts (for histones; Abcam protocol) or 10 μg of nuclear extracts (for nonhistone proteins; prepared according to Ambrosino et al., 2010) were resolved by SDS-PAGE and blotted on nitrocellulose membranes. Membranes were blocked for 1h in TBS-Tween containing 5% milk and incubated overnight at 4°C with the indicated antibodies diluted in TBS-Tween containing 3% milk. After washes, the membranes were incubated with secondary antibodies diluted in TBS-Tween containing 3% milk for 1h at room temperature. HRP conjugates were detected with enhanced chemiluminescence (ECL Plus, Amersham Biosciences). The following primary antibodies were used for immunoblotting: H3K4me3 (Diagenode pAb-MEHAHS-024, A1-010, 1:1000); H3K27me3 (Millipore 07-449, DAM-1588246, 1:1000); H4 (Abcam, ab7311, 826236, 1:1000); Ezh2 (Active Motif 39639, 23809001, 1:1000); Suz12 (Abcam ab12073-100, 418328, 1:500); β-actin (Abcam ab16039, 104192, 1:500), p16 (M-156, sc-1207, H1810), p19_ARF (5-C3-1, sc-32748, A0411), p21 (F-5, sc-6246, G0210). Secondary antibodies used: HRP-conjugated polyclonal swine anti-rabbit IgG (Dako, P0399, 00042894, 1:4000); HRP-conjugated polyclonal rabbit anti-mouse IgG (Dako, P0161, 00046035, 1:3000); HRP-conjugated polyclonal rabbit anti-rat IgG (Dako, P0450, 00017777, 1:1000).

Immunofluorescent Stainings

Cells were fixed in 4% formaldehyde, permeabilized with 0.1% Triton X-100 and blocked with 3% donkey serum. Overnight incubation was performed with Oct3/4 (Santa Cruz sc-5279, c-20), Klf4 (R&D Systems AF3158) or Nanog (E-biosciences 14-5761-80) antibody at 4°C. Alexa Fluor 647 donkey anti-goat or anti-mouse IgG were used as secondary antibodies.

Clonogenic Assays

After sorting, ES cells were plated in serum or 2i + LIF at clonal density in duplicate wells (800 cells per well of a 6-well dish). Colonies were grown for 5 days in serum or for 7 days in 2i + LIF. Alkaline phosphatase staining was performed to score for colonies consisting of largely undifferentiated cells (undiff), mixed, and largely differentiated (diff) cells.

Cell Sorting (FACS)

Cell sorting was performed according to Wray et al. (2011).

Details of Sequencing

Quality control of DNA libraries prepared for sequencing was made by qPCR and by running the products on a Bioanalyzer (BioRad). Samples were sequenced to a depth of approximately 20 million uniquely mapped tags per sample. Sequences were aligned to the mouse MM9 reference genome with the Illumina Analysis Pipeline allowing one mismatch. Only the tags aligning to one position on the genome were considered for further analysis. For RNA-seq, further analysis was performed with the 36 bp aligned sequence

reads. For ChIP-seq, identical sequence tags were discarded to obtain a nonredundant set, and the 36 bp sequence reads were directionally extended to 300 bp, corresponding to the length of the original fragments used for sequencing. The output data were converted to Browser Extensible Data (BED) files for downstream analysis and Wiggle (WIG) files for viewing.

Identification and Quantification of ncRNAs

For de novo identification of ncRNAs of the strand-specific RNA-seq, signals on the minus and plus strand were analyzed separately. Signals were quantified in 10 kb bins at a genome-wide scale. Bins that overlapped with known coding or noncoding RNAs (present in RefSeq, GenBank, or ENSEMBL) were excluded for further analysis. Subsequently, we selected bins with signals above background. Signals were averaged for TNGA 2i ES cells and E14 cells adapted to 2i (8 passages), as well as for E14 serum ES cells and TNGA cells adapted to serum (8 passages). Known ncRNAs were selected from the RefSeq database. RNAs were considered to be differential whether there was at least a 2-fold difference between the 2i and serum conditions, with an extra constraint set by a p value < 0.2 (student t test).

Peak Calling

Identification of the H3K27me3 binding sites (peak calling) was performed via FindPeaks (Fejes et al., 2008) with a loose FDR cut-off of $< 1 \times 10^{-2}$, subpeaks 0.9, triangles distribution and duplicate filter. The number of tags per peak was normalized for the genomic length of the peak (expressed as reads/kb), by which the peaks were categorized (Figure 4A). Overlaps with genomic features were determined with Galaxy (main.g2.bx.psu.edu).

ChIP-Seq Repeat Analysis

For the repeat analysis of ChIP-seq profiles, the mappings were performed with the maq (mapping and assembly with qualities) aligner version 0.7.1 (Li et al., 2008). The major advantage for the repeat analysis as compared to the ELAND Pipeline is that, if a sequenced read aligns on multiple places on the genome, maq assigns it randomly at one of these positions. This is useful when studying repeat classes, as the reads representing these classes will by definition map on multiple genomic locations. However, these will almost exclusively belong to the same class of repeat. All reads mapped by maq were included in downstream analyses. To enable direct comparisons, the samples were ratio normalized for the total number of tags mapped by maq. Sequence coordinates of various repeat classes were downloaded from the UCSC Table Browser (RepMask 3.2.7; rmskRM327). ChIP-seq tags were considered to represent a repeat class in case of any overlap of the 36 nt sequenced fragment with the repeat class. Subsequently, the number of ChIP-seq tags representing a repeat class was counted.

SUPPLEMENTAL REFERENCES

Ambrosino, C., Tarallo, R., Bamundo, A., Cuomo, D., Franci, G., Nassa, G., Paris, O., Ravo, M., Giovane, A., Zambrano, N., et al. (2010). Identification of a hormone-regulated dynamic nuclear actin network associated with estrogen receptor alpha in human breast cancer cell nuclei. Mol. Cell. Proteomics *9*, 1352–1367.

Fejes, A.P., Robertson, G., Bilenky, M., Varhol, R., Bainbridge, M., and Jones, S.J. (2008). FindPeaks 3.1: a tool for identifying areas of enrichment from massively parallel short-read sequencing technology. Bioinformatics *24*, 1729–1730.

Li, H., Ruan, J., and Durbin, R. (2008). Mapping short DNA sequencing reads and calling variants using mapping quality scores. Genome Res. *18*, 1851–1858.

Morris, D.P., Michelotti, G.A., and Schwinn, D.A. (2005). Evidence that phosphorylation of the RNA polymerase II carboxyl-terminal repeats is similar in yeast and humans. J. Biol. Chem. *280*, 31368–31377.

Yuan, P., Han, J., Guo, G., Orlov, Y.L., Huss, M., Loh, Y.H., Yaw, L.P., Robson, P., Lim, B., and Ng, H.H. (2009). Eset partners with Oct4 to restrict extraembryonic trophoblast lineage potential in embryonic stem cells. Genes Dev. *23*, 2507–2520.

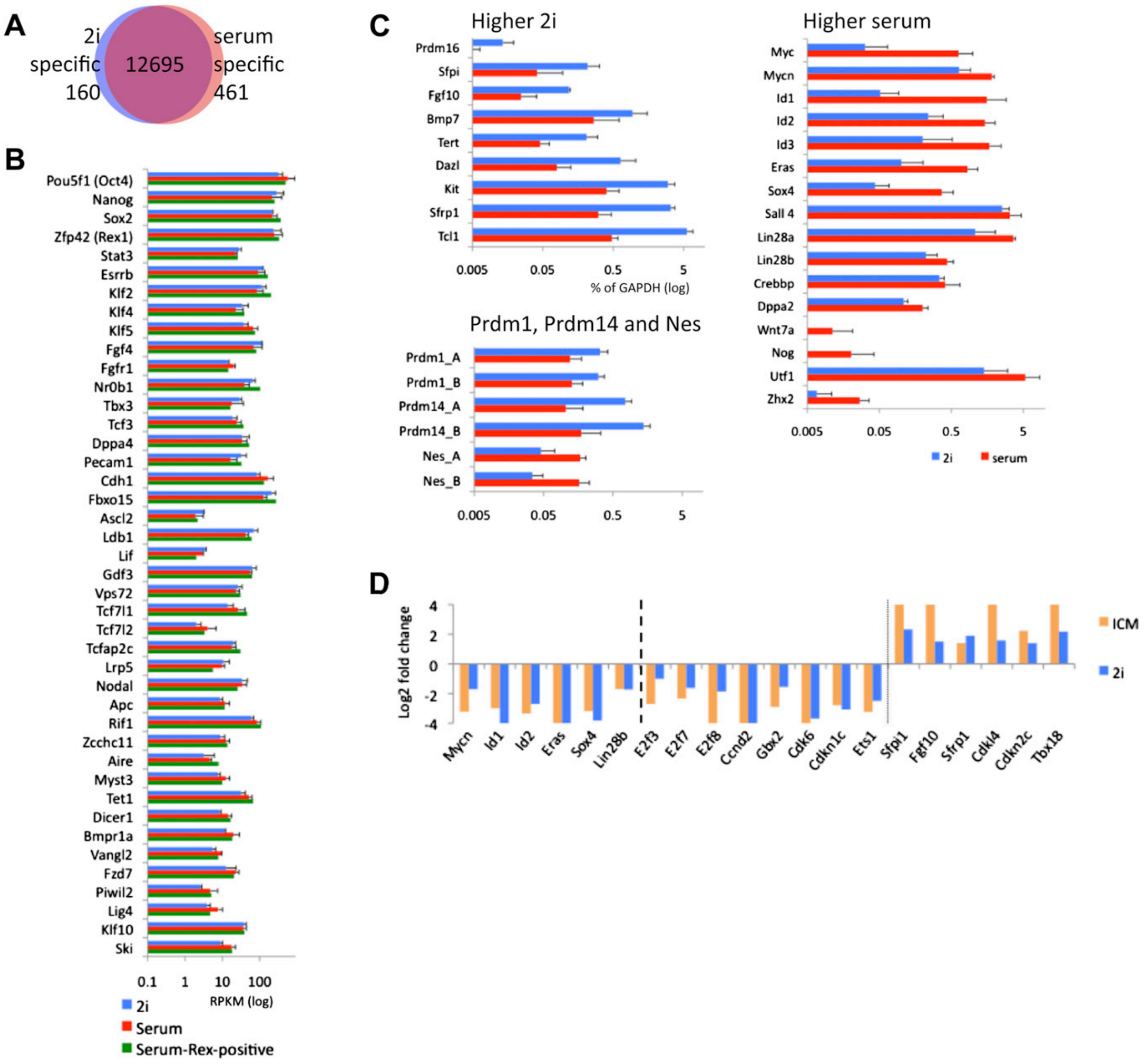

Figure S1. Transcriptome Profiling of ES Cells in 2i and Serum, Related to Figure 1

(A) Venn-diagram of genes specific for 2i (below detection limit in serum), present in both 2i and serum, or only present in serum (below detection limit in 2i).

(B) Addition to Figure 1B: Expression of all Stem Cell Maintenance (SCM) genes, as annotated by Gene Ontology, with similar expression in 2i and serum.

(C) RT-qPCR validation for all differential SCM genes shown in Figure 1B, and for *Prdm1*, *Prdm14*, and *Nes* (with two independent primer pairs, A and B respectively).

(D) RNA expression of differential SCM genes, differential cell-cycle control regulators and other key transcription factors in the Inner Cell Mass (ICM) versus serum (Tang et al., 2010; orange) and 2i versus serum (this study; blue). Shown is the fold change (in $\log_2$ values) as compared to serum ES cells.

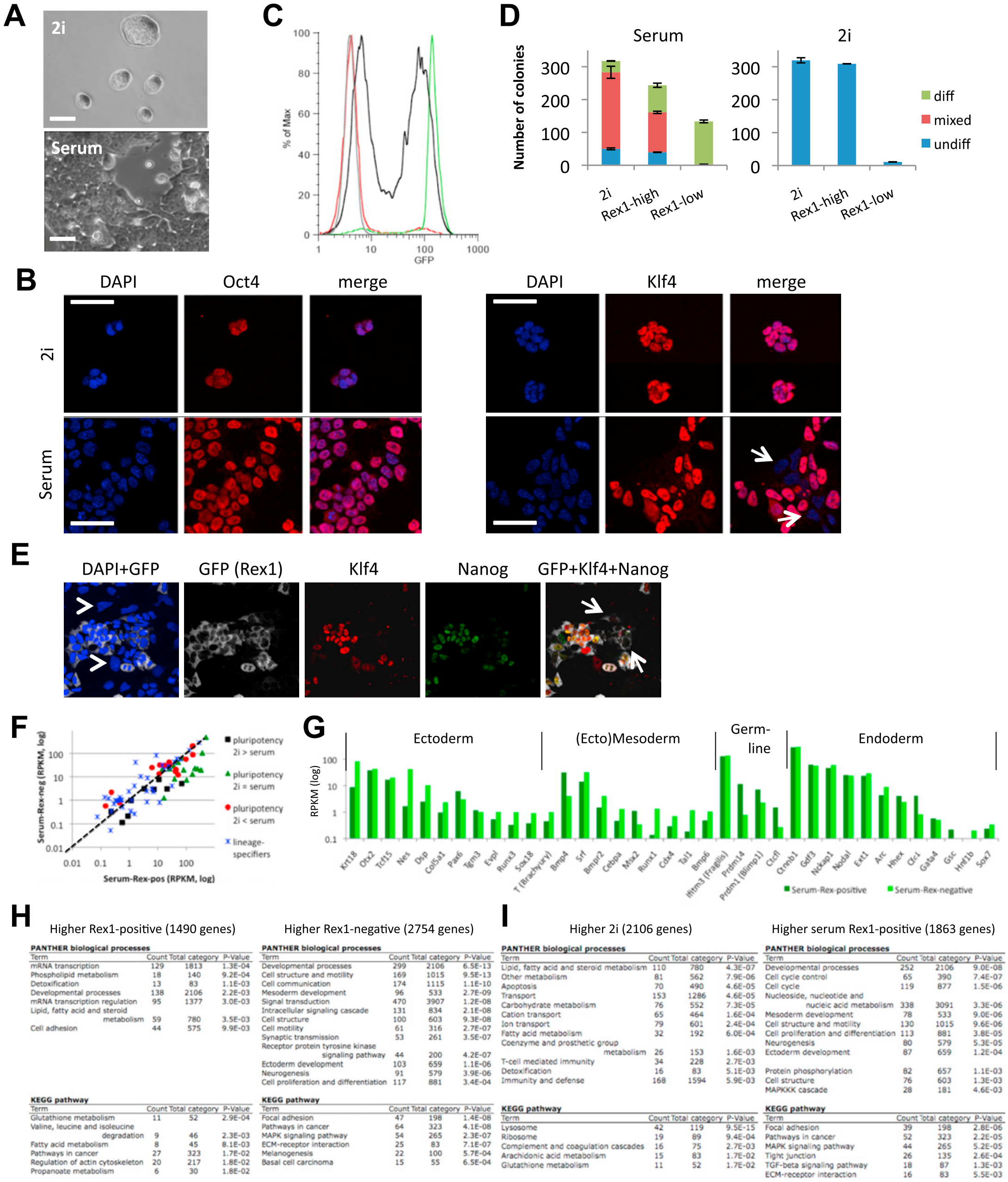

Figure S2. Analysis of Rex1-Positive and -Negative Serum ES Cell Fractions, Related to Figure 1

(A) ES cell morphology in 2i or serum culture.

(B) Oct4 (left) and Klf4 (right) immunostaining of ES cells in 2i or serum. Arrows point to Klf4-negative ES cells in serum.

(C) GFP flow cytometry profile of the total population of Rex1-GFP serum ES cells (black) and ES cells without any GFP transgene (negative control, gray (largely overlapping red)). The GFP-negative and -positive sorted Rex1-GFP serum ES cells used in this study are shown in red and green, respectively.

(D) Number of colonies after re-plating 2i ES cells and Rex1-positive and Rex1-negative ES cells at clonal density in serum (left) or 2i (right). After 5 days (serum) or 7 days (2i + LIF), alkaline phosphatase staining was performed to discriminate between colonies consisting of largely undifferentiated cells (undiff), mixed, or largely differentiated (diff) cells.

(E) Klf4 and Nanog immunostaining of unsorted Rex1-GFP serum ES cells. The arrowheads in the DAPI stainings point to Rex1-, Klf4-, and Nanog-negative cells, the arrows in the GFP+Klf4+Nanog stainings to Rex1-positive, but Klf4- and Nanog-negative cells.

(F) Comparison of expression of pluripotency and lineage-specific genes (as shown in Figures 1B and 1D) of Rex1-positive (Serum-Rex-pos) and Rex1-negative (Serum-Rex-neg) ES cells.

(G) Transcript levels of genes associated with the various germ layers in Rex1-positive and Rex1-negative serum ES cells.

(H) Functional analysis of the differential genes between Rex1-positive and Rex1-negative serum ES cells.

(I) Functional analysis of the differential genes between 2i ES cells and Rex1-positive serum ES cells.

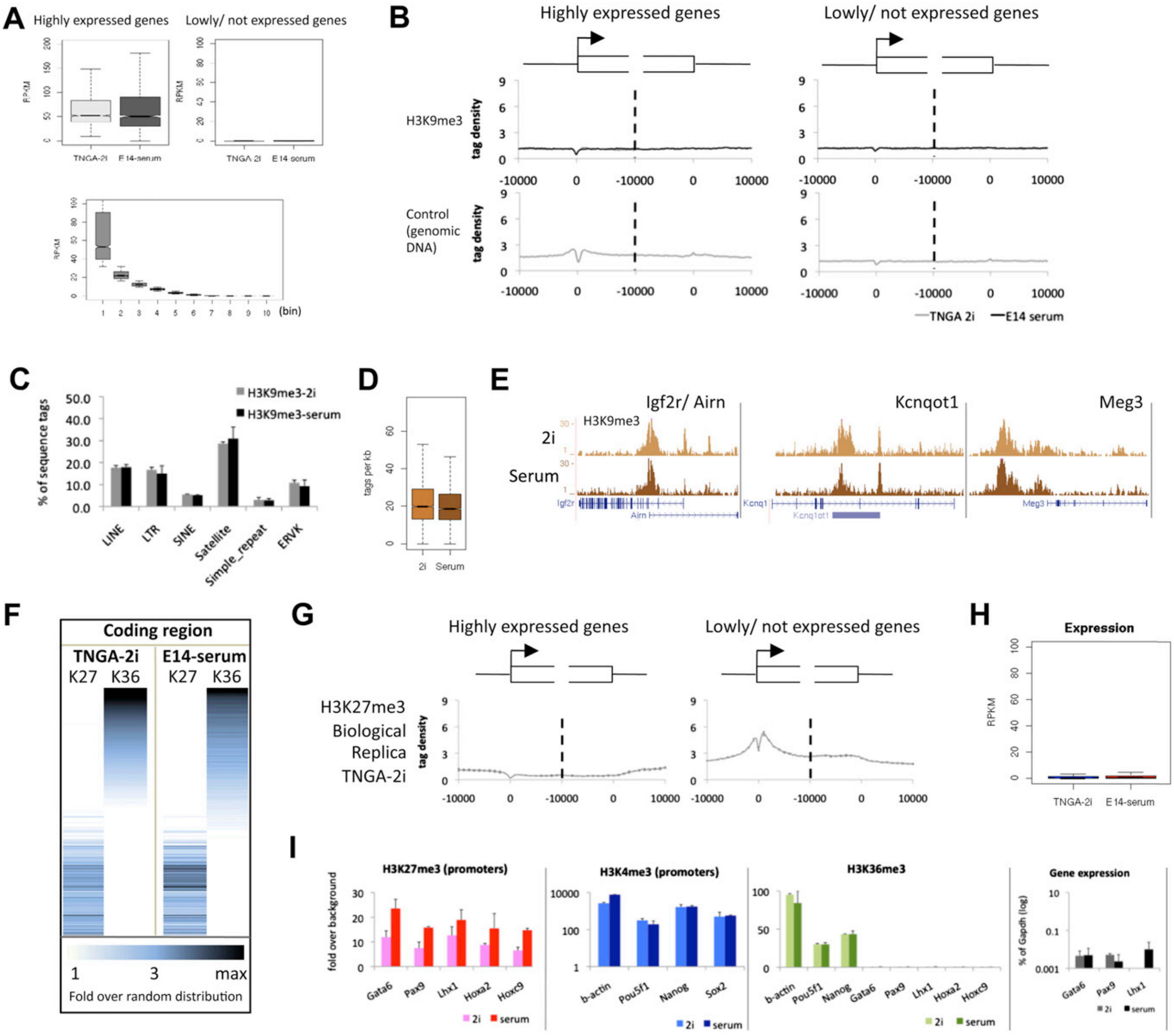

Figure S3. H3K27me3 Is Greatly Diminished at Promoters of Silent Genes and at Hox Clusters in ES Cells in 2i, Related to Figure 3

(A) Expression levels of the two groups of genes shown in Figure 3A. For comparative purposes, the expression level of all genes equally divided into ten bins according to expression level is shown at the bottom.

(B) Similar to Figures 3A and 3B: Average H3K9me3 profiles over active and inactive genes, as well as the negative control (genomic DNA derived from the chromatin input).

(C) Percentage of H3K9me3 tags present in the major repeat categories.

(D) Quantification of the H3K9me3 enriched genomic loci, as determined by Yuan et al. (2009), in 2i and serum.

(E) Typical examples of H3K9me3, enrichment over three imprinted genes.

(F) H3K27me3 (“K27”) and H3K36me3 (“K36”) intensity plots for the genomic coding regions of all genes (ranked by TNGA-2i H3K36me3 values), showing that H3K27me3 and H3K36me3 are mutual exclusive.

(G) Similar to Figure 3B: H3K27me3 profiling of biological replica of TNGA in 2i.

(H) Expression of the genes shown in Figure 3F. See Figure S3A (bottom) for a binned overview for the expression level of all genes.

(I) Validation of the ChIP-seq and RNA-seq profiles by ChIP-qPCR or RT-qPCR, respectively.

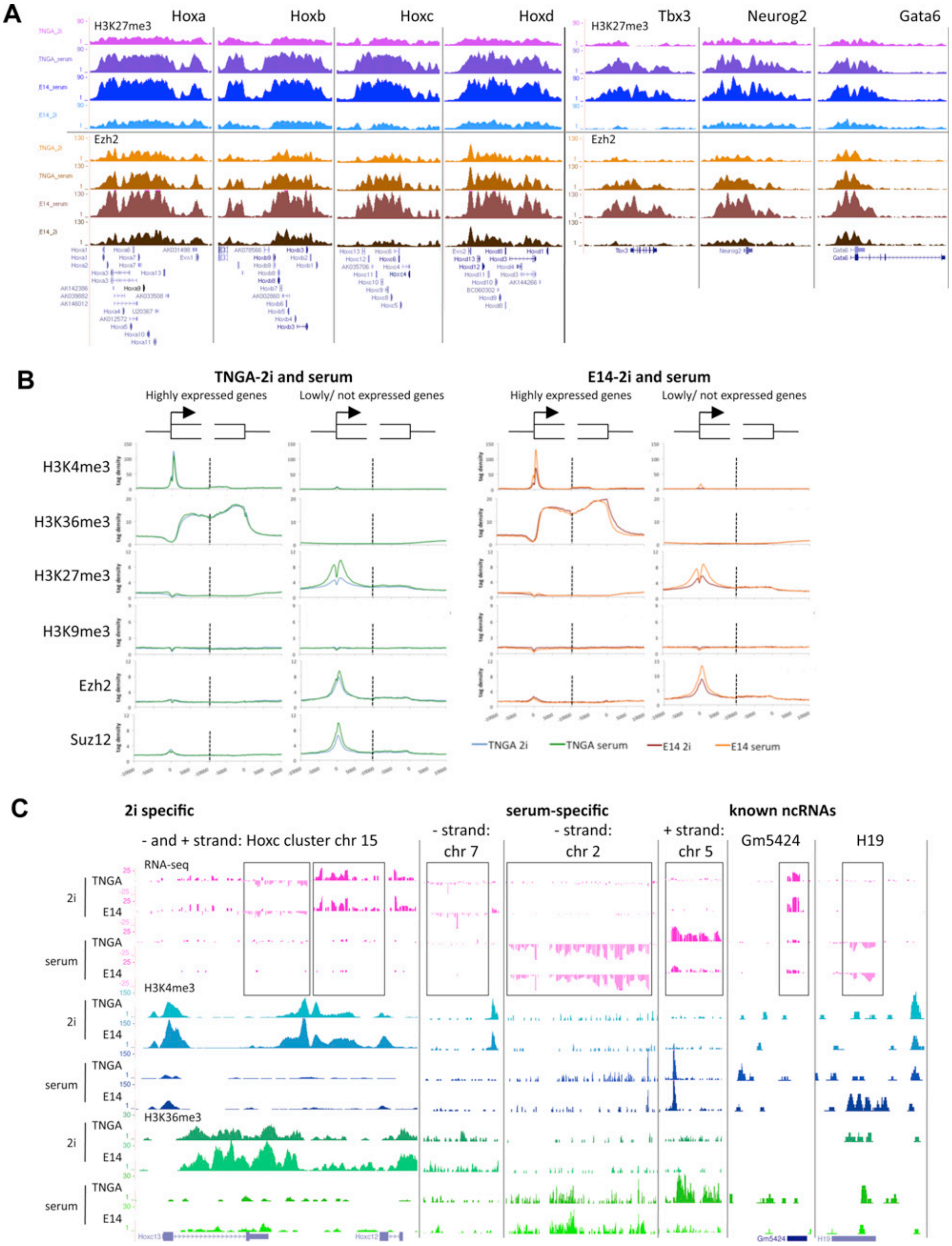

Figure S4. Interconvertibility of the Epigenome between ES Cells in 2i and Serum, Related to Figure 4

(A) Typical examples of the interconvertibility of the H3K27me3 and Ezh2 epigenome in 2i and serum ES cells.

(B) Average H3K4me3, H3K36me3, H3K27me3. H3K9me3, Ezh2 and Suz12 epigenetic profiles at 2000 most active (left plots) and 2000 silent (right plots) genes around the 5′ and 3′ end, in TNGA cells (left; TNGA 2i: maintained and derived in 2i; TNGA serum; adapted to serum) and E14 cells (right; E14 serum: maintained and derived in serum; E14 2i: adapted to 2i).

(C) UCSC genome browser examples of known and previously unidentified ncRNA, identified and quantified with strand-specific rRNA depleted RNA-seq. RNA-seq signals on the + strand have positive values (dark pink), signals on the - strand have negative values (light pink). Differential expression of these genes is clearly supported by the H3K4me3 and H3K36me3 epigenome profiles.

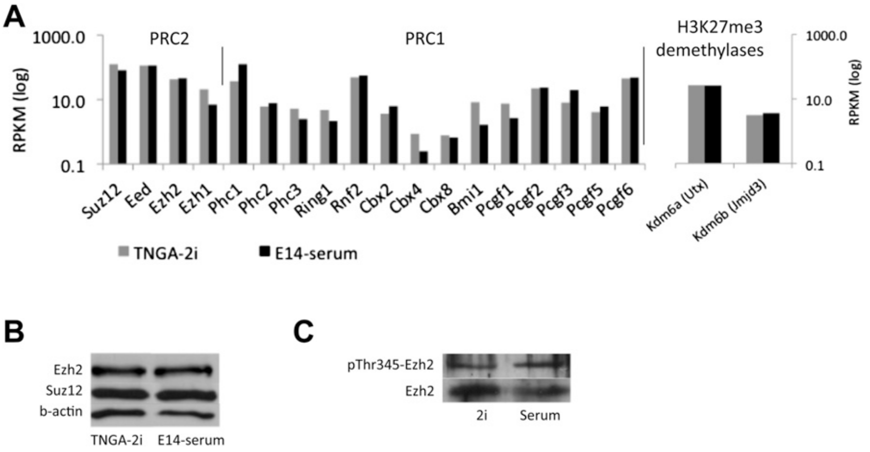

Figure S5. H3K27me3 Deposition, Related to Figure 4

(A) Expression levels of PRC2 and PRC1 subunits, and H3K27me3 demethylases, as determined by RNA-seq.

(B and C) Immunoblot analysis of PRC2 subunits (B) and pThr345-Ezh2 (C).

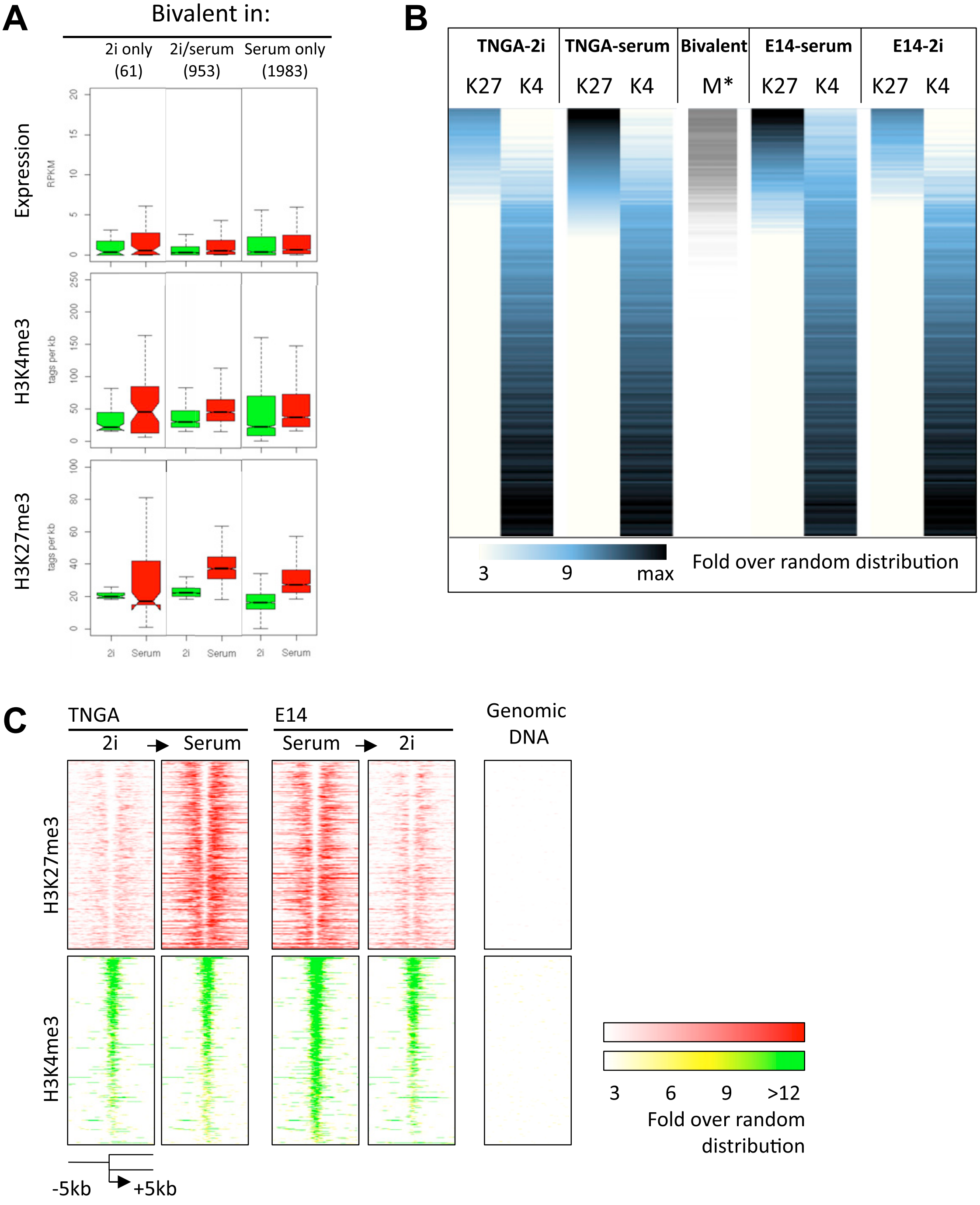

Figure S6. Bivalency, Related to Figure 5

(A) Expression levels and H3K4me3/ H3K27me3 levels of genes bivalent in 2i, serum or both.

(B and C) Bivalent marks are interconvertible between 2i and serum conditions. H3K27me3 and H3K4me3 intensity plots at the promoters of all genes similar to Figure 5A, but including the E14 cells (B). H3K27me3 and H3K4me3 intensity plots of all promoters that are bivalent in TNGA serum with a similar setup as Figure 5C but including the E14 cells (C).

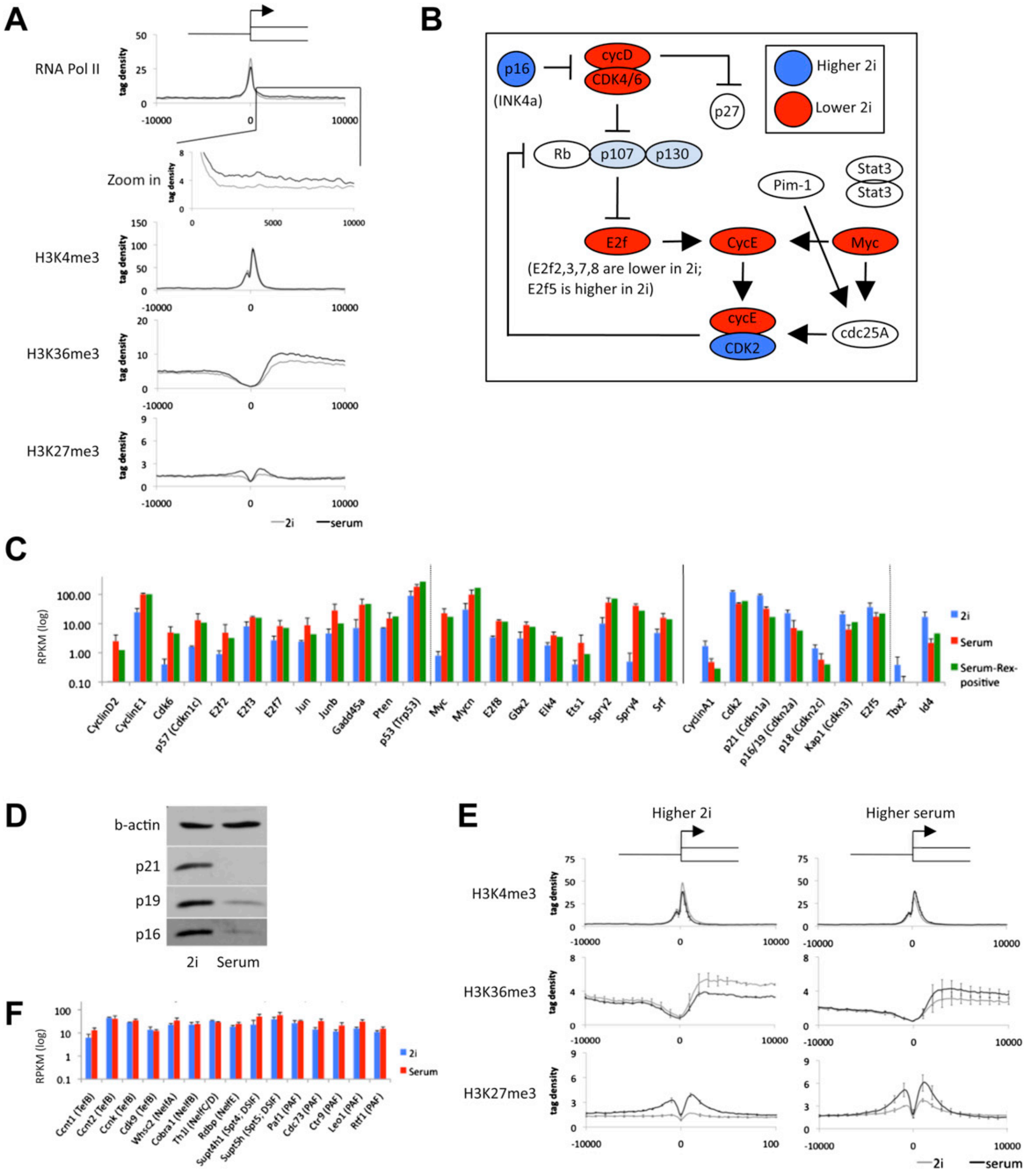

Figure S7. RNA Polymerase II Pausing in Naive ES Cells, Related to Figure 6

(A) Addition to Figure 6A: Averaged RNA Polymerase II, H3K4me3, H3K36me3 and H3K27me3 profiles at promoters of Myc target genes upregulated in serum.

(B) Gene expression as determined by RNA-seq of genes involved in the typical G1/S checkpoint network (after Burdon et al. (2002)).

(C) Gene expression of the major cell-cycle regulators with differential gene expression between cells grown in 2i and serum. Left: Genes higher in serum; Right: Genes higher in 2i. The expression values of Rex1-positive serum ES cells are also included.

(D) Immunoblot analysis for p16 (INK4a), p19 (ARF) and p21.

(E) Averaged H3K4me3, H3K36me3 and H3K27me3 profiles of the promoter region of genes that are more highly expressed in 2i (left) or serum (right). Profiles for TNGA 2i ES cells and E14 2i adapted ES cells are combined to generate average values and error bars (2i), the same was performed for E14 serum ES cells and the TNGA serum adapted ES cells (serum).

(F) RNA-seq expression levels of regulators of pausing and pause release (TefB, NELF, DSIF, PAF) in 2i and serum.
